# Supplementary material for: Women’s decision-making autonomy and utilisation of maternal healthcare services: results from the Bangladesh Demographic and Health Survey
Source: BMJ Open. 2017 Sep 7;7(9):e017142. doi: 10.1136/bmjopen-2017-017142 (PMC5595179; doi:10.1136/bmjopen-2017-017142)
Supplement: Author's manuscript [file bmjopen-2017-017142.draft_revisions.pdf]

BMJ Open is committed to open peer review. As part of this commitment we make the peer review history of every article we publish publicly available.

When an article is published we post the peer reviewers' comments and the authors' responses online. We also post the versions of the paper that were used during peer review. These are the versions that the peer review comments apply to.

The versions of the paper that follow are the versions that were submitted during the peer review process. They are not the versions of record or the final published versions. They should not be cited or distributed as the published version of this manuscript.

BMJ Open is an open access journal and the full, final, typeset and author-corrected version of record of the manuscript is available on our site with no access controls, subscription charges or pay-per-view fees (http://bmjopen.bmj.com).

If you have any questions on BMJ Open's open peer review process please email editorial.bmjopen@bmj.com

Women’s decision making autonomy and utilization of maternal healthcare services: Bangladesh Demographic and Health Survey

Journal:	*BMJ Open*
Manuscript ID	bmjopen-2017-017142
Article Type:	Research
Date Submitted by the Author:	04-Apr-2017

Complete List of Authors:	Ghose, Bishwajit; School of Medicine and Health Management, Tongji Medical College, Huazhong University of Science and Technology<br>Tang, Shangfeng; School of medicine and health management, Tongji medical college, Huazhong university of science and technology, Health management<br>Yaya, Sanni; School of International Development and Global Studies, University of Ottawa.<br>He, Zhifei; School of Medicine and Health Management, Tongji Medical College, Huazhong University of Science & Technology, Department of Health Administration<br>Ghosh, Sharmistha; University of Dhaka<br>Feng, Zhanchun; Health Manament

**Primary Subject Heading**:	Health services research
Secondary Subject Heading:	Public health
Keywords:	Bangladeshi women, Decision-making autonomy, Maternal healthcare services, Spousal cooperation

SCHOLARONE™ Manuscripts

For peer review only

Women's decision making autonomy and utilization of maternal healthcare services: Bangladesh Demographic and Health Survey

Ghose Bishwajit[1] Shangfeng Tang[1] Sanni Yaya[2]

Zhifei He[1] Sharmistha Ghosh[3] Zhanchun Feng[1]

[1] School of Medicine and Health Management, Tongji Medical College, Huazhong University of Science and Technology, Wuhan, China.

[2]School of International Development and Global Studies, University of Ottawa. Canada.

[3]Department of Sociology, University of Dhaka, Dhaka 1000, Bangladesh

Email addresses:

Ghose Bishwajit : brammaputram@gmail.com

Shangfeng Tang: sftang@hust.edu.cn

Sanni Yaya : sanni.yaya@uOttawa.ca

Zhifei He : houis123@163.com

Sharmistha Ghosh : aimidu.hust@yahoo.com

Zhanchun Feng :zcfeng@hust.edu.cn

Correspondence: Shangfeng Tang, sftang@hust.edu.cn

School of Medicine and Health Management, Tongji Medical College, Huazhong University of Science and Technology, Hangkong Road 13, Wuhan, Hubei, 430030 People's Republic of China; Email: sftang@hust.edu.cn

For peer review only

Abstract:

Objectives: The aim of this study was to determine the association between women's decision-making power and utilization of maternal healthcare services among Bangladeshi women.

Settings: This is a nationally representative survey that encompassed Dhaka, Rajshahi, Rangpur, Chittagong, Khulna, Barisal and Sylhet in Bangladesh. Sample households were selected by a two-stage stratification technique. Firstly, 207 clusters in urban areas and 393 in rural areas were selected for 600 enumeration areas with proportional probability. In the second stage, on average 30 households were selected systematically from the enumeration areas. Finally 17,989 household were selected for the survey of which 96% were interviewed successfully.

Participants: Cross-sectional data on 4309 non-pregnant women were collected from Bangladesh demographic and health survey 2014. Decision making status on respondent's own health care, large household purchases, having a say on child's health care, and visiting to family or relatives were included in the analysis.

Results: Prevalence of at least four antenatal attendance, facility delivery and post-natal check-up were respectively 32.6% (95%CI=31.2- 34), 40.6% (95%CI=39.13- 42.07) and 66.3% (95%CI=64.89- 67.71). Compared to women who could make decisions alone, women the urban areas who had to decide on their healthcare with husband/partner had 20% (95%CI=0.794-1.799) higher and those in rural areas had 35% (95%CI=0.464-0.897) lower odds of attending at least four antenatal visits. Women in urban and rural areas had respectively 43% (95%CI=0.941-2.169) and 28% (95%CI=0.928-1.751) higher odds of receiving post-natal check-up when made their health decisions jointly with husband/partner.

Conclusion: Neither making decisions alone, nor deciding jointly with husband/partner was always positively associated with the utilization of all three types MHS. This study concludes that better spousal cooperation on household and health issues could lead higher utilization of MHS services.

Key terms: Bangladeshi women; Decision-making autonomy; Maternal healthcare services; Spousal cooperation.

For peer review only - http://bmjopen.bmj.com/site/about/guidelines.xhtml

Strengths and limitations

1. This is one of the few studies focusing on the correlation between women's decision making autonomy and maternal healthcare utilization in a South Asian country.
2. Based on data from Bangladesh Demographic and Health Survey 2014, this study provides the most recent scenario of the utilization of three key important components of maternal healthcare.
3. Regional differences were observed in the prevalence of decision making autonomy and utilization of maternal healthcare services. However, there association no strong indication regarding the importance of decision making autonomy for the uptake of maternal healthcare.
4. The survey was cross-sectional. Therefore, it cannot affirm any causal inference or direction of the association.

Introduction

There is a widespread consensus regarding the pivotal role of utilization of maternal healthcare services (MHS) on reducing maternal and child mortality and promoting women's reproductive health. Maternal mortality refers to deaths caused by pregnancy or childbirth related complications. Since 2015, global maternal mortality rate (MMR) dropped by 44% at an average annual decline of 2.3%, however still remains the leading cause of death among adult women ageing between 15 and 49 years[1]. The burden of maternal mortality is also disproportionately skewed towards the developing countries[2.] The most important causes of maternal mortality in developing countries are unsafe abortion, haemorrhage, eclampsia and obstructed labour as they together account for nearly two-third of total maternal mortality globally[3,4]. Growing consensus suggests that a vast majority of these deaths are actually preventable simply by adopting the necessary precautions provisioned through basic maternal healthcare services (MHS)[3,5].

For peer review only - http://bmjopen.bmj.com/site/about/guidelines.xhtml

Burden of maternal mortality is also historically high in Bangladesh. However, the country has achieved noteworthy progress in terms of reducing MMR by a three quarter by 2015 as a part of its meeting the Millennium Development Goal 5A (MDG)[6]. According to a study based on Bangladesh Maternal Mortality Surveys (BMMS), maternal mortality was the largest single cause of death (20%) for women aged 15–49 followed by malignancy and infectious diseases, and ranked third a decade later (14% deaths)[7]. Despite the continued progress, the country is lagging far behind ensuring universal access to reproductive health (MDG 5B) and the rate of utilisation of the basic MHS at national level remains quite low[8]. According to Bangladesh Demographic and Health Survey (BDHS 2007), only about half of all mothers attended one or more antenatal visit and about one-fifth received at least one postnatal service. Mothers who do not attend ANC services are also more unlikely to deliver at health facilities and receive postnatal services[9], which increases the risk of pregnancy and childbirth related complications[5,9,10]. Rate of health facility delivery is also notably low in Bangladesh with three-quarters of all births occurring at home and merely one-fifth are attended by a skilled birth attendant (SBA)[10], which is far below the internationally agreed target (90% births to be attended by SBA by 2015) [11].

Previous studies have attempted to explore the barriers to utilization of maternal healthcare services, some from demographic, economic[9,12-13] and some from sociocultural and behavioral perspectives[9,10,14-15]. Apart from the socioeconomic aspects, there is also a growing number of study emphasizing the role of women's decision-making autonomy on maternal health service utilization and pregnancy outcomes[16,17]. However, the results remain somewhat mixed as some researches stress on the importance of wife's autonomy on making decisions and some proposing that joint decision-making by husbands/partners and wives can produce better reproductive health outcomes than when one partner is left behind from decision-making tasks. In the perspective of Bangladesh however, involvement of husbands/partners in decision-making is particularly important because most families are male-headed and it is also the male figures who usually play the dominant role in important household decision making such as income expenditure, healthcare movement[17]. In the South Asian countries including Bangladesh, gender discrimination and inequality remains a widespread phenomenon across various walks of life such as decision making autonomy, intrahousehold resource allocation, property rights, access to healthcare[18,19]. Women's autonomy is a multidimensional concept which is hard to represent in a single definition. In short, it conveys a set of discrete components or phenomena essential for ensuring that women can exercise their rights with full potential. Therefore, the aim of this study was to determine the association between women's decision-making power and utilization of maternal healthcare services among Bangladeshi women. For, this study, women's decision-

For peer review only - http://bmjopen.bmj.com/site/about/guidelines.xhtml

making autonomy was measured across four different themes ranging having a say on their own and children's healthcare decisions to household purchases and visiting family and relatives. Data were sourced from the latest BDHS survey which provides a large scale quality data and representative of the general population.

Methods

The survey: BDHS 2014

This is a cross-sectional study based on data from the Bangladesh demographic and health survey conducted in 2014. The 2014 survey was the sixth to be conducted in the country. This is a nationally representative survey that included both urban and rural areas encompassing all seven administrative divisions—Dhaka, Rajshahi, Rangpur, Chittagong, Khulna, Barisal and Sylhet. A division is a collection of districts (zilas), and each district is further divided into administrative units (upazilas), which are further divided into urban and rural areas. Sample households were selected by a two-stage stratification technique. Firstly, 207 clusters in urban areas and 393 in rural areas were selected for 600 enumeration areas with proportional probability. In the second stage, on average 30 households were selected systematically from the enumeration areas. Finally 17,989 household were selected for the survey of which 96% were interviewed successfully. Details on the survey and sampling technique are available in the final report.

Variables

Outcome variable: The outcome variables chosen for this study were three basic types of maternal healthcare services offered by the healthcare system in Bangladesh: 1) Antenatal care services, 2) facility delivery services, and 3) Post-delivery checkup services. Information on these topics were collected by face to face interview with the respondents. Women were asked the number of times they received Antenatal care (ANC), and the frequency ranged from ''0'' to ''20''. However, for this study ANC was categorized as adequate (4/4+) and inadequate (<4) as per WHO recommendation which suggests at least four ANC attendance during pregnancy. Place of delivery was categorized as 'Facility delivery' and 'Delivery at home'. Facility delivery included delivery in public or private hospitals or clinics, NGO clinics. The third outcome variable- Post-delivery checkup services, was categorized as yes (For those who received any postnatal checkup) and no (For those who did not receive any postnatal checkup).

For peer review only - http://bmjopen.bmj.com/site/about/guidelines.xhtml

Explanatory variables of interest were women decision making power on following four themes: 1. Person who usually decides on respondent's health care, 2. Person who usually decides on large household purchases, 3. Final say on: Child health care, 4. Person who usually decides on visits to family or relatives. In types of decision making tasks a joint decision by women and their husband was highest. Possible answers were Respondent alone, Respondent and Husband/Partner jointly, Husband/Partner alone, and Other. The categories were collapsed into three by combining the last two into one (Husband/partner alone and Other).

The covariates included in the analysis were Age: 15-20/21-24/25-29/30+; Educational attainment: No education/Incomplete primary/Complete primary/Incomplete secondary/Complete secondary/Higher; Currently working: No/Yes; Wealth index: Poorer/Middle/Richer/Richest/Poorest; Parity: 1/2/3/3+.

Data analysis

Datasets were checked for missing values and outliers and weighted prior to analysis. Basic sociodemographic variables were described by descriptive statistics. Chi-square bivariate tests were performed to examine the group differences (Utilization vs Non- utilization of MCHs) for all the explanatory variables. The variables that showed significance at $p<=0.25$ in the bivariate tests were retained for final regression analysis. The association between utilization of MCHs and the independent variables was measured by binary logistic regression. Three separate regression models were run for each of the outcome variables. Results of the regression analysis were presented as adjusted odds ratios with corresponding 95% confidence intervals. The outcomes of the regression analysis were reported in terms of adjusted odds ratios (AOR) and corresponding 95% confidence intervals. Model fitness was verified by the Hosmer-Lemeshow goodness-of-fit test. All tests were two-tailed and was considered significant at 5%. Data were analysed using SPSS® version 22.

Ethics

All participants gave informed consent prior to taking part in the voluntary interview. The survey was approved by the ICF International Institutional Review Board (IRB) who is responsible for reviewing the procedures and questionnaires for standard DHS surveys.

Results

Population characteristics

For peer review only - http://bmjopen.bmj.com/site/about/guidelines.xhtml

Table 1 shows that the participant were almost evenly distributed across the four age groups with lowest frequency for 30+. About one-third women were from rural areas which is similar to the country level scenario. 13.3% of the women no formal education and 11.4% had completed primary. Rate of illiteracy was high among rural women compared to urban counterparts (9.8 Vs 14.9%). Rate of completion of secondary was 7.5% and 11.8% had higher than secondary level education. Only about one-fifth of the women reported having an employment, and urban women had slightly higher rate of employment (22.9 Vs 19.0%) than rural women. Majority of the women belonged to the highest wealth quintile (20.5%) and little less than one-fifth in the poorest wealth quintile (18.9%). A wide wealth disparity was observed between participants in urban and rural areas as 43.2% of the women in the highest wealth quintile were from urban areas compared with only 9.2% from rural areas. Two-fifth of the women had only one child and 14.2% had more than three children.

Table 1: Basic characteristics of the study population, BDHS 2014.

**Variables**	**N (%)**	**Urban**<br>1381 (32.5)	**Rural**<br>2873 (67.5)
**Age**			
15-20	1178 (27.7)	25.3	28.8
21-24	1144 (26.9)	28.7	26.0
25-29	1091 (25.6)	27.1	25.0
30+	841 (19.8)	18.9	20.2
**Educational attainment**			
No education	564 (13.3)	9.8	14.9
Incomplete primary	658 (15.5)	12.8	16.7
Complete primary	487 (11.4)	10.2	12.0
Incomplete secondary	1724 (40.5)	38.6	41.5
Complete secondary	319 (7.5)	8.9	6.8
Higher	502 (11.8)	19.6	8.0
**Currently working**			
No	3333 (78.3)	81.0	77.1
Yes	921 (21.7)	19.0	22.9
**Wealth index**			
Poorer	806 (18.9)	8.6	26.2

For peer review only - http://bmjopen.bmj.com/site/about/guidelines.xhtml

Middle	814 (19.1)	7.8	24.3
Richer	901 (21.2)	12.0	22.6
Richest	860 (20.2)	28.4	17.7
Poorest	873 (20.5)	43.2	9.2
**Parity**			
1	1700 (40.0)	44.6	37.7
2	1286 (30.2)	32.2	29.3
3	664 (15.6)	13.5	16.6
3+	604 (14.2)	9.7	16.4

For peer review only

Based on the availability of on the dataset, four types of decision making tasks were considered relevant to MCH in this study: 1. Person who usually decides on respondent's health care, 2. Person who usually decides on large household purchases, 3. Final say on: Child health care, 4. Person who usually decides on visits to family or relatives. For all types of decision making tasks a joint decision by women and their husband was highest. Table 2 shows that frequency of having autonomy in all types of the decisions was lower among rural women except for final say on child's healthcare. In majority of the cases decisions were made jointly by women and the husband/partner. Husbands/partners had notably higher rate of autonomy than women in making these decisions in both in rural and urban areas.

Table 2: Women's household decision-making characteristics, BDHS 2014.

**Types of decision making**	**Respondent alone**		**Respondent & husband/partner**		**Husband/partner alone/other**	
	**Urban**	**Rural**	**Urban**	**Rural**	**Urban**	**Rural**
**Person who usually decides on respondent's health care**	12.2	12	51.3	47.2	36.5	40.9
**Person who usually decides on large household purchases**	7.1	5.4	53.4	46.5	39.5	48.0
**Final say on: Child's health care**	14.2	15.2	58.8	54.2	27.0	30.6

For peer review only - http://bmjopen.bmj.com/site/about/guidelines.xhtml

**Person who usually decides on visits to family or relatives**	8.3	7.4	53.4	47.3	38.3	45.2

Table 3 shows the prevalence of availing the three types of maternal healthcare services stratified by place of residency. Prevalence of ANC attendance, facility delivery and post-natal check-up were respectively 32.6%, 40.6% and 66.3% (Not shown in the table). Results of cross-tabulation show that the rate of utilization of these services were higher among urban women compared to their rural counterparts, higher among women ageing between 21-24 years, having incomplete secondary level schooling, living in the richest households, currently working and had given birth only once. In majority of the cases women who could make the decisions jointly with husband/partner were more like to enjoy the MCH services.

Table 3: **Percentage of women who reported utilizing three types of MCH across the explanatory variables, BDHS 2014.**

	**ANC**		**Facility delivery**		**Health check-up after birth**	
	**Urban (46.1)**	**Rural (26)**	**Urban (42.1)**	**Rural (67.7)**	**Urban (79.4)**	**Rural (60.0)**
**Age**	23.4	30.1	23.0	30.1	24.4	30.0
15-20	29.0	26.7	28.2	27.1	29.4	26.0
21-24	27.9	27.4	29.2	25.2	27.6	24.4
25-29	19.6	15.8	19.6	17.5	18.7	19.5
30+	23.4	30.1	23.0	30.1	24.4	30.0
P-value	0.478	**0.005**	**0.051**	**0.101**	0.322	0.291
**Educational attainment**						
No education	5.2	6.8	5.0	6.8	8.6	10.9
Incomplete primary	7.7	12.2	9.3	10.1	11.5	14.7
Complete primary	7.4	8.6	6.3	9.7	8.8	11.3
Incomplete secondary	36.1	47.9	39.8	45.3	38.6	43.2
Complete secondary	11.0	10.7	11.1	10.9	9.9	8.6
Higher	32.7	13.9	28.5	17.2	22.7	11.4

For peer review only - http://bmjopen.bmj.com/site/about/guidelines.xhtml

P-value	**<0.001**	**<0.001**	**<0.001**	**<0.001**	**<0.001**	**<0.001**
**Wealth index**						
Poorest	5.2	15.6	3.1	12.6	5.7	20.0
Poorer	4.4	19.7	5.8	17.4	6.3	21.9
Middle	7.4	21.9	8.1	25.3	10.5	24.2
Richer	25.0	25.9	25.8	25.9	28.0	21.2
Richest	58.1	16.8	57.2	18.7	49.5	12.8
P-value	**<0.001**	**<0.001**	**<0.001**	**<0.001**	**<0.001**	**<0.001**
**Currently working**						
No	82.1	79.0	83.6	81.1	81.7	74.8
Yes	17.9	21.0	16.4	18.9	18.3	25.2
P-value	0.336	**0.157**	**0.004**	**<0.001**	**0.204**	**<0.001**
**Parity**						
1	50.1	44.0	50.2	49.1	46.3	42.2
2	34.7	32.1	33.0	29.2	33.3	28.7
3	10.0	14.7	11.0	13.8	12.4	15.5
4	5.2	9.2	5.8	8.0	8.0	13.7
P-value	**<0.001**	**<0.001**	**<0.001**	**<0.001**	**<0.001**	**<0.001**
**Decides on own health care**						
Alone	11.8	15.0	13.1	11.8	12.2	12.0
Jointly	53.7	49.3	51.8	49.6	52.6	48.1
Husband/other	34.5	35.7	35.0	38.5	35.1	39.9
P-value	**0.246**	**<0.001**	0.291	**0.165**	**0.096**	0.418
**Decides on large household purchases**						
Alone	7.8	6.8	8.1	5.5	7.7	5.8
Jointly	55.6	46.9	52.3	47.6	53.8	47.5
Husband/other	36.6	46.3	39.5	46.9	38.5	46.7
P-value	**0.108**	**0.117**	**0.189**	**0.144**	**0.149**	**0.006**
**Decides on Child health care**						
Alone	13.8	17.8	15.0	16.3	14.6	16.2
Jointly	61.9	55.1	59.4	54.9	59.4	54.9

For peer review only

For peer review only - http://bmjopen.bmj.com/site/about/guidelines.xhtml

Husband/other	24.3	27.1	25.5	28.8	26.0	28.9
P-value	**0.077**	**0.015**	**0.175**	**0.219**	**0.231**	**0.035**
**Decides on visits to family or relatives**						
**Alone**	8.6	8.0	9.9	7.2	8.3	7.7
**Jointly**	57.5	46.7	53.6	47.9	54.3	47.2
**Husband/other**	33.9	45.3	36.5	44.9	37.4	45.1
P-value	**0.007**	**0.140**	**0.029**	**0.102**	**0.075**	**0.031**

Association between decision making ability and utilization of MCH

Results of regression analysis on the association between decision making ability and utilization of MCH are presented in table 4.

In the urban areas, women who could decide their healthcare with husband/partner had 20% (95%CI=0.794-1.799) higher odds of attending at least four antenatal care compared to those who made could make decisions alone. In the rural areas however, women who could make decisions alone were 35% (95%CI=0.464 0.897) less likely to do so. The odds of delivering at a health facility were 25% (95%CI=0.888-1.748) higher among rural women who made own health decisions jointly with husband/partner. Women in urban and rural areas had respectively 43% (0.941-2.169) and 28% (0.928-1.751) higher odds of receiving post-natal check-up when made their health decisions jointly with husband/partner. Women who had less autonomy on deciding large household purchases were respectively 28% (95%CI=0.384-1.365) and 20% (95%CI=0.492-1.285) less likely to have at least 4 ANC visits. Rural women who had to decide on large household purchases with husband/partner had 15% (95%CI=0.547-1.332) lower odds of receiving post-natal check-up.

Having autonomy on deciding children's health care did not show noticeable impact on receiving ANC services. Odds of receiving post-natal check-up were respectively 22% (95%CI=0.503-1.212) and 31% (95%CI=0.501-0.946) lower and facility delivery respectively 11% (95%CI=0.574-1.413) and 12% (95%CI=0.577-1.266) lower among urban and rural women who had to make the decisions jointly with husband/partner. In urban areas, women who did not have the autonomy to decide on visiting to family or relatives alone were 18% (95%CI=0.491-1.362) less likely to attend at least four antenatal visits. The odds of receiving post-natal check-up were

For peer review only - http://bmjopen.bmj.com/site/about/guidelines.xhtml

respectively 32% (95%CI=0.760-2.311) and 11% higher among urban and women who could to decide on visiting to family or relatives jointly with husband/partner.

Table 4: Association between decision making ability and utilization of MCH in Bangladesh, BDHS 2014.

	Antenatal care OR (95%CI)		Delivery at a health facility OR (95%CI)		Health check-up after delivery OR (95%CI)	
	Urban	Rural	Urban	Rural	Urban	Rural
**Decides on own health care**						
Alone	1	1	1	1	1	1
Jointly	1.195 (0.794-1.799)	0.645 (0.464 0.897)	0.996 (0.606-1.327)	1.246 (0.888-1.748)	1.428 (0.941-2.169)	1.275 (0.928-1.751)
Husband/other	1.087 (0.806-1.750)	0.983 (0.635-1.227)	1.072 (0.731-1.572)	1.001 (0.703-1.424)	1.052 (0.662-1.671)	1.035 (0.815-1.579)
**Decides on large household purchases**						
Alone	1	1	1	1	1	1
Jointly	0.724 (0.384-1.365)	0.795 (0.492-1.285)	1.050 (0.621-1.776)	0.997 (0.629-1.581)	1.02 (0.251-1.745)	0.854 (0.547-1.332)
Husband/other	0.970 (0.361-1.444)	0.805 (0.493-1.315)	0.734 (0.420-1.282)	0.924 (0.587-1.455)	0.950 (0.617-1.935)	0.943 (0.548-1.497)
**Decides on Child health care**						
Alone	1	1	1	1	1	1
Jointly	0.978 (0.641-1.491)	0.983 (0.558-1.499)	0.897 (0.574-1.413)	0.884 (0.577-1.266)	0.781 (0.503-1.212)	0.688 (0.501-0.946)
Husband/other	1.100 (0.751-1.612)	0.930 (0.696-1.243)	1.079 (0.713-1.635)	0.945 (0.620-1.452)	0.898 (0.562-1.384)	0.949 (0.645-1.617)
**Decides on visits to family or**						

For peer review only

For peer review only - http://bmjopen.bmj.com/site/about/guidelines.xhtml

**relatives**						
Alone	1	1	1	1	1	1
Jointly	0.818 (0.491-1.362)	1.018 (0.645-1.727)	0.981 (0.664-0.178)	1.108 (0.776-1.729)	1.325 (0.760-2.311)	1.113 (0.757-1.636)
Husband/other	1.063 (0.701-1.928)	1.078 (0.709-1.640)	0.887 (0.421-1.121)	1.075 (0.711-1.625)	1.050 (0.819-1.567)	0.943 (0.645-1.378)

N.B. Adjusted for the variables found significant in the bivariate test in table 3.

For peer review only

Discussion and conclusion

Main findings

Based on a nationally representative data from Bangladesh demographic and health survey, this study explored the association between women's decision-making power and utilization of antenatal care, facility delivery and post-natal health check-up among adult non-pregnant women ageing between 15 and 49 years in Bangladesh. Our results show that Prevalence of antenatal care attendance, facility delivery and post-natal check-up were respectively 32.6%, 40.6% and 66.3% which indicates a considerable improvement compared to the previous estimates. In urban and rural areas respectively, the rate of attending at least four antenatal visits increased from 36.7% and 11.7% in 2004 (44.8% and 19.8% in 2011) to 46.1% and 26% in 2014[20]. Utilisation of health facility delivery increased from 12 % in 2004 (>29 % in 2011) to >40% in 2014[21], and postnatal check-up of mothers increased from 27.3% to >66% during the same period[22].

Compared to women who decided on their health care alone, those who decided jointly with husband/partner had higher likelihood of utilizing all three types of services (except for antenatal visits among rural women). However, women could decide large household purchases alone had higher likelihood of attending at least four antenatal visits. Similar association was observed for utilization of post-natal care among women in rural but not urban areas. Having decision-making autonomy on child's healthcare showed significant association with the utilization of facility delivery and postnatal check-ups but not antenatal visits. Having decision-making autonomy on visiting family/relatives showed significant association with the utilization of postnatal check-ups but not antenatal visits and facility delivery.

For peer review only - http://bmjopen.bmj.com/site/about/guidelines.xhtml

Comparison with existing literature

Results indicate that in majority of the cases decisions were made jointly followed by men alone and women alone. A previous study one conducted on south Asian countries reported a similar situation that women's healthcare decision were made without their participation in Nepal (72.7%), Bangladesh (54.3%) and India (48.5%) [23]. Regarding the association between decision-making autonomy and MHS utilization, comparison between the findings of the present study with the existing ones requires consideration of several important issues. Firstly, different studies uses different indicators of women's decision-making autonomy and different types of MHS. Moreover, in some studies report involvement of various family members and not just women and husbands/partners. Regardless of that, our findings have consistent and conflicting points with previous ones. Low level of women's autonomy was found to be a contributing factor to poor maternal health service utilization in Nepal[24], India[25] , but not in Kenya[26]. In Ethiopia, decision-making autonomy on place of birth by showed a positive association with utilization of institutional delivery[27].

While women's lack of decision-making autonomy can be attributed to poor utilization of MHS, it however should not be ignored that autonomy in certain circumstances can also result in less spousal communication and low male-involvement in reproductive care. Growing number of studies indicate that inadequate spousal communication and male-involvement in reproductive care are associated with poor reproductive and sexual health consequences, and recommend policies to promote spousal communication and cooperation for improved maternal health outcomes[28,29]. In Nepal for instance, economic autonomy among women was associated with lower likelihood of couple communication during pregnancy, while domestic decision-making autonomy was associated with both lower likelihood of intra-spousal communication during pregnancy and husband's presence at antenatal visits [30]. Husbands' involvement in antenatal care has been shown to have a positive influence on utilization of antenatal visits in Ethiopia[27]. Husbands' involvement was also associated with utilization of professional care during delivery in rural Bangladesh and India [31].

In light of the abovementioned discussion, it is suggestible health projects targeting to improve the utilization of MHS should try to focus on women's autonomy and at the same time promote male involvement in women's reproductive care. A qualitative study on male participation in reproductive health in Bangladesh reported poor interaction between husband and wife regarding sexual reproductive health issues which makes it difficult for men to recognise the reproductive health issues of women[32]. The study also reported that men do not feel comfortable to take their

For peer review only - http://bmjopen.bmj.com/site/about/guidelines.xhtml

wives to the health facility, which suggests the presence of complex social and cultural factors preventing effective spousal communication regarding reproductive health issues. In the traditionally male-dominated society in Bangladesh where male figures are usually involved in family decision making, excluding men from maternal health decision making issues could prevent men from informed decision making for their wives/partners.

This study has several limitations to declare. First, this study included only four aspects to of women's decision making. Thus, the findings do no indicate women's overall mobility and empowerment but rather specifically focus on a limited range of indicators. As the participants were only women, there remain a potential for biasness/discordance regarding the level of autonomy enjoyed by women as this is to a large extent a subjective phenomenon. Arguably, collecting information from both men and women could generate more reliable picture on women's mobility and empowerment. So the association between women's autonomy and health-care-service use may be underestimated when only women's reports are considered[30]. In addition, spousal autonomy is a complex concept and difficult to quantify and there is no universally agreed definition or tool for measurement. Last but not least, utilization status of MHS was reported by women and was not verified from medical records, and therefore subject to recall bias.

Abbreviations: BDHS: Bangladesh Demographic and Health Survey; MHS: Maternal health service utilization; MMR: Maternal mortality rate; SBA: Skilled birth attendant.

Conflict of Interest: None to declare.

Funding: This research was supported by the National Natural Science Foundation of China (71473097).

Data availability: Data are available from DHS website through registration.

Acknowledgement: Authors would like to express sincere thanks to DHS for distributing the datasets that made this study possible.

Authors' contributions: SFT, BG and ZCF designed the study.BG and SFT conducted the analysis and wrote the paper. SY, HZF, SG participated in editing of the paper. All authors discussed the results and approved the revision of the final manuscript.

For peer review only

References

1. Benedict OA, Kontie MM, Martin S, *et al*. Distribution of causes of maternal mortality among different socio-demographic groups in Ghana; a descriptive study. *BMC Public Health* 2011; 11: 159.
2. Rush D. Nutrition and maternal mortality in the developing world. *Am J Clin Nutr* 2000;72(1):(suppl) 212S-240S.
3. Lale S, Chou D, Gemmill A, et al. Global causes of maternal death: a WHO systematic analysis. *Lancet Global Health* 2014;2:e323–33.
4. Kassebaum NJ, Bertozzi-Villa A, Coggeshall MS, et al. Global, regional, and national levels and causes of maternal mortality during 1990–2013: a systematic analysis for the Global Burden of Disease Study 2013. *Lancet* 2014; 384:980–1004.
5. Yaya S, Bishwajit G, Shah V. Wealth, education and urban–rural inequality and maternal healthcare service usage in Malawi. *BMJ Global Health* 2016; 1(2): e000085.
6. Marge K, Iqbal A, Malay KM, *et al*. Reducing Maternal Mortality and Improving Maternal Health: Bangladesh and MDG 5. *J Health Popul Nutr* 2008; 26(3): 280–294.
7. Quamrun N, Shams EA, Kanta J, *et al*. Causes of adult female deaths in Bangladesh: findings from two National Surveys. *BMC Public Health* 2015; 15: 911.
8. Shahabuddin AS, Delvaux T, Abouchadi S, *et al*. Utilization of maternal health services among adolescent women in Bangladesh: A scoping review of the literature. *Trop Med Int Health 2015*; 20(7):822-9.
9. Christiana RT, Cynthia LH, Peter H, *et al*. Why don't some women attend antenatal and postnatal care services? a qualitative study of community members' perspectives in Garut, Sukabumi and Ciamis districts of West Java Province, Indonesia. *BMC Pregnancy Childbirth* 2010; 10: 61.
10. Nazrul I, Mohammad TI, Yukie Y. Practices and determinants of delivery by skilled birth attendants in Bangladesh. *Reprod Health* 2014; 11: 86.
11. Sonya Crowe, Martin Utley, Anthony Costello and Christina Pagel. How many births in sub-Saharan Africa and South Asia will not be attended by a skilled birth attendant between 2011 and 2015? BMC Pregnancy and Childbirth, 2012, 12:4.
12. McNamee P, Ternent L, Hussein J. Barriers in accessing maternal healthcare: evidence from low-and middle-income countries. *Expert Rev Pharmacoecon Outcomes Res* 2009;9(1):41-8.

For peer review only - http://bmjopen.bmj.com/site/about/guidelines.xhtml

13. Ruhul A, Nirali MS, Stan B. Socioeconomic factors differentiating maternal and child health-seeking behavior in rural Bangladesh: A cross-sectional analysis. *Int J Equity Health* 2010; 9: 9.

14. Krishna KD, Yuba RP, Resham BK, *et al*. Barriers to Utilization of Antenatal Care Services in Eastern Nepal. *Front Public Health* 2015; 3: 197

15. David OA, Olufemi TO, Marianne V, *et al*. Determinants of health care seeking behaviour during pregnancy in Ogun State, Nigeria. *Reprod Health* 2016; 13(1): 32.

16. John KG, Bernard O, Alexander YS, *et al.* How intra-familial decision-making affects women's access to, and use of maternal healthcare services in Ghana: a qualitative study. *BMC Pregnancy Childbirth* 2015; 15: 173.

17. Story WT, Burgard SA. Couples' reports of household decision-making and the utilization of maternal health services in Bangladesh. *Soc Sci Med* 2012; 75(12):2403-11.

18. Fikree FF, Pasha O. Role of gender in health disparity: the South Asian context. *British Medical Journal* 2004; 328(7443):823–826.

19. Bishwajit G, Sarker S, Yaya S. Socio-cultural aspects of gender-based violence and its impacts on women's health in South Asia. *F1000Research* 2016; 5:802

20. Mohammad HP, Md NS, Jalal U, Ulrika E. Progress in the utilization of antenatal and delivery care services in Bangladesh: where does the equity gap lie? *BMC Pregnancy Childbirth* 2016; 16: 200.

21. Kamal SM. Preference for institutional delivery and caesarean sections in Bangladesh. *J Health Popul Nutr* 2013; 31(1):96-109.

22. Uzma S, Asiruddin Sk, Helal, *et al*. Immediate and Early Postnatal Care for Mothers and Newborns in Rural Bangladesh. *J Health Popul Nutr* 2006; 24(4): 508–518.

23. Senarath U, Gunawardena NS. Women's autonomy in decision making for health care in South Asia. *Asia Pac J Public Health* 2009; 21(2):137-43.

24. Ramesh Adhikari. Effect of Women's autonomy on maternal health service utilization in Nepal: a cross sectional study. *BMC Womens Health* 2016; 16: 26.

25. Mistry R, Galal O, Lu M. Women's autonomy and pregnancy care in rural India: a contextual analysis. *Soc Sci Med* 2009; 69(6):926-33.

26. Fotso JC, Ezeh AC, Essendi H. Maternal health in resource-poor urban settings: how does women's autonomy influence the utilization of obstetric care services? *Reprod Health* 2009; 16:6-9.

For peer review only - http://bmjopen.bmj.com/site/about/guidelines.xhtml

27. Biratu BT, Lindstrom DP. The influence of husbands' approval on women's use of prenatal care: results from Yirgalem and Jimma towns, south west Ethiopia. *Ethiopian Journal of Health Development* 2006; 20: pp. 84–92.
28. Mullany BC, Becker S, Hindin MJ. The impact of including husbands in antenatal health education services on maternal health practices in urban Nepal: results from a randomized controlled trial. *Health Education Research* 2007; 22: pp. 166–176.
29. Rasch V, Lyaruu MA. Unsafe abortion in Tanzania and the need for involving men in postabortion contraceptive counselling. *Studies in Family Planning* 2005; 36: pp. 301–310.
30. Allendorf K. Couples' reports of women's autonomy and health-care use in Nepal. *Stud Fam Plann* 2007; 38(1):35-46.
31. Deependra KT, Anke N. Women's autonomy and husbands' involvement in maternal health care in Nepal. *Social Science & Medicine* 2013; 93(9):1–10.
32. Shahjahan M, Kabir M. Why males in Bangladesh do not participate in reproductive health: lessons learned from focus group discussions. *Int Q Community Health Educ* 2006; 26(1):45-59.

For peer review only

STROBE 2007 (v4) Statement—Checklist of items that should be included in reports of *cross-sectional studies*

Section/Topic	Item #	Recommendation	Reported on page #
**Title and abstract**	1	(*a*) Indicate the study's design with a commonly used term in the title or the abstract	2
		(*b*) Provide in the abstract an informative and balanced summary of what was done and what was found	2
**Introduction**			
Background/rationale	2	Explain the scientific background and rationale for the investigation being reported	3
Objectives	3	State specific objectives, including any prespecified hypotheses	4
**Methods**			
Study design	4	Present key elements of study design early in the paper	5
Setting	5	Describe the setting, locations, and relevant dates, including periods of recruitment, exposure, follow-up, and data collection	5
Participants	6	(*a*) Give the eligibility criteria, and the sources and methods of selection of participants	5
Variables	7	Clearly define all outcomes, exposures, predictors, potential confounders, and effect modifiers. Give diagnostic criteria, if applicable	5
Data sources/ measurement	8*	For each variable of interest, give sources of data and details of methods of assessment (measurement). Describe comparability of assessment methods if there is more than one group	5,6
Bias	9	Describe any efforts to address potential sources of bias	*15*
Study size	10	Explain how the study size was arrived at	5
Quantitative variables	11	Explain how quantitative variables were handled in the analyses. If applicable, describe which groupings were chosen and why	6
Statistical methods	12	(*a*) Describe all statistical methods, including those used to control for confounding	6
		(*b*) Describe any methods used to examine subgroups and interactions	6
		(*c*) Explain how missing data were addressed	6
		(*d*) If applicable, describe analytical methods taking account of sampling strategy	
		(*e*) Describe any sensitivity analyses	
**Results**			

For peer review only

Participants	13*	(a) Report numbers of individuals at each stage of study—eg numbers potentially eligible, examined for eligibility, confirmed eligible, included in the study, completing follow-up, and analysed	5
		(b) Give reasons for non-participation at each stage	5
		(c) Consider use of a flow diagram	
Descriptive data	14*	(a) Give characteristics of study participants (eg demographic, clinical, social) and information on exposures and potential confounders	6-7
		(b) Indicate number of participants with missing data for each variable of interest	
Outcome data	15*	Report numbers of outcome events or summary measures	8
Main results	16	(*a*) Give unadjusted estimates and, if applicable, confounder-adjusted estimates and their precision (eg, 95% confidence interval). Make clear which confounders were adjusted for and why they were included	11-12
		(*b*) Report category boundaries when continuous variables were categorized	11-12
		(*c*) If relevant, consider translating estimates of relative risk into absolute risk for a meaningful time period	
Other analyses	17	Report other analyses done—eg analyses of subgroups and interactions, and sensitivity analyses	
**Discussion**			
Key results	18	Summarise key results with reference to study objectives	2
Limitations	19	Discuss limitations of the study, taking into account sources of potential bias or imprecision. Discuss both direction and magnitude of any potential bias	15
Interpretation	20	Give a cautious overall interpretation of results considering objectives, limitations, multiplicity of analyses, results from similar studies, and other relevant evidence	
Generalisability	21	Discuss the generalisability (external validity) of the study results	
**Other information**			2
Funding	22	Give the source of funding and the role of the funders for the present study and, if applicable, for the original study on which the present article is based	15

*Give information separately for cases and controls in case-control studies and, if applicable, for exposed and unexposed groups in cohort and cross-sectional studies.

Note: An Explanation and Elaboration article discusses each checklist item and gives methodological background and published examples of transparent reporting. The STROBE checklist is best used in conjunction with this article (freely available on the Web sites of PLoS Medicine at http://www.plosmedicine.org/, Annals of Internal Medicine at http://www.annals.org/, and Epidemiology at http://www.epidem.com/). Information on the STROBE Initiative is available at www.strobe-statement.org.

For peer review only

Women's decision making autonomy and utilization of maternal healthcare services: Bangladesh Demographic and Health Survey

Journal:	*BMJ Open*
Manuscript ID	bmjopen-2017-017142.R1
Article Type:	Research
Date Submitted by the Author:	09-Jun-2017
Complete List of Authors:	Ghose, Bishwajit; School of Medicine and Health Management, Tongji Medical College, Huazhong University of Science and Technology<br>Feng, Da; School of Medicine and Health Management, Tongji Medical College, Huazhong University of Science & Technology, Department of Health Administration<br>Tang, Shangfeng; School of medicine and health management, Tongji medical college, Huazhong university of science and technology, Health management<br>Yaya, Sanni; School of International Development and Global Studies, University of Ottawa.<br>He, Zhifei; School of Medicine and Health Management, Tongji Medical College, Huazhong University of Science & Technology, Department of Health Administration<br>Ghosh, Sharmistha; University of Dhaka<br>Udenigwe, Ogochukwu; School of International Development and Global Studies, University of Ottawa.<br>Feng, Zhanchun; Health Manament
**Primary Subject Heading**:	Health services research
Secondary Subject Heading:	Public health
Keywords:	Bangladeshi women, Decision-making autonomy, Maternal healthcare services, Spousal cooperation

Women's decision making autonomy and utilization of maternal healthcare services: Bangladesh Demographic and Health Survey

Ghose Bishwajit[1†] Da Feng[1†] Shangfeng Tang[1*] Sanni Yaya[2]

Zhifei He[1] Ogochukwu Udenigwe[2] Sharmistha Ghosh[3] Zhanchun Feng[1]

[1] School of Medicine and Health Management, Tongji Medical College, Huazhong University of Science and Technology, Wuhan, China.

[2]School of International Development and Global Studies, University of Ottawa. Canada.

[3]Department of Sociology, University of Dhaka, Dhaka 1000, Bangladesh

Email addresses:

Ghose Bishwajit: brammaputram@gmail.com

Da Feng: fdnunu@163.com

Shangfeng Tang: sftang@hust.edu.cn

Sanni Yaya : sanni.yaya@uOttawa.ca

Zhifei He: houis123@163.com

Ogochukwu Udenigwe: ocudenigwe@gmail.com

Sharmistha Ghosh : aimidu.hust@yahoo.com

Zhanchun Feng :zcfeng@hust.edu.cn

† Both authors contributed equally to this work

***Correspondence:** Shangfeng Tang, sftang@hust.edu.cn

School of Medicine and Health Management, Tongji Medical College, Huazhong University of Science and Technology, Hangkong Road 13, Wuhan, Hubei, 430030 People's Republic of China; Email: sftang@hust.edu.cn

For peer review only

For peer review only - http://bmjopen.bmj.com/site/about/guidelines.xhtml

Abstract:

Objectives: The aim of this study was to determine the association between women's decision-making power and utilization of maternal healthcare services among Bangladeshi women.

Settings: This is a nationally representative survey that encompassed Dhaka, Rajshahi, Rangpur, Chittagong, Khulna, Barisal and Sylhet in Bangladesh. Sample households were selected by a two-stage stratification technique. Firstly, 207 clusters in urban areas and 393 in rural areas were selected for 600 enumeration areas with proportional probability. In the second stage, on average 30 households were selected systematically from the enumeration areas. Finally 17,989 household were selected for the survey of which 96% were interviewed successfully.

Participants: Cross-sectional data on 4309 non-pregnant women were collected from Bangladesh demographic and health survey 2014. Decision making status on respondent's own health care, large household purchases, having a say on child's health care, and visiting to family or relatives were included in the analysis.

Results: Prevalence of at least four antenatal attendance, facility delivery and post-natal check-up were respectively 32.6% (95%CI=31.2- 34), 40.6% (95%CI=39.13- 42.07) and 66.3% (95%CI=64.89- 67.71). Compared to women who could make decisions alone, women in the urban areas who had to decide on their healthcare with husband/partner had 20% (95%CI=0.794-1.799) higher odds of attending at least four antenatal visits and those in rural areas had 35% (95%CI=0.464-0.897) lower odds of attending at least four antenatal visits. Women in urban and rural areas had respectively 43% (95%CI=0.941-2.169) and 28% (95%CI=0.928-1.751) higher odds of receiving post-natal check-up when their health decisions were made jointly with their husband/partner.

Conclusion: Neither making decisions alone, nor deciding jointly with husband/partner was always positively associated with the utilization of all three types of MHS. This study concludes that better spousal cooperation on household and health issues could lead to higher utilization of MHS services.

Key terms: Bangladeshi women; Decision-making autonomy; Maternal healthcare services; Spousal cooperation.

For peer review only

Strengths and limitations

1. This is one of the few studies focusing on the correlation between women's decision making autonomy and maternal healthcare utilization in a South Asian country.
2. Based on data from Bangladesh Demographic and Health Survey 2014, this study provides the most recent scenario of the utilization of three key important components of maternal healthcare.
3. Regional differences were observed in the prevalence of decision making autonomy and utilization of maternal healthcare services. However, the association was not a strong indication regarding the importance of decision making autonomy for the uptake of maternal healthcare.
4. The survey was cross-sectional. Therefore, it cannot affirm any causal inference or direction of the association.

Introduction

There is a widespread consensus regarding the pivotal role of the utilization of maternal healthcare services (MHS) in reducing maternal and child mortality and promoting women's reproductive health. Maternal mortality refers to deaths caused by pregnancy or childbirth related complications. Since 2015, global maternal mortality rate (MMR) dropped by 44% at an average annual decline of 2.3%, however, it still remains the leading cause of death among adult women ageing between 15 and 49 years[1]. The burden of maternal mortality is also disproportionately skewed towards the developing countries[2.] The most important causes of maternal mortality in developing countries are unsafe abortion, haemorrhage, eclampsia and obstructed labour as they together account for nearly two-thirds of total maternal mortality globally[3,4]. Growing consensus suggests that a vast majority of these deaths are actually preventable simply by adopting the necessary precautions provisioned through basic maternal healthcare services (MHS)[3,5].

The burden of maternal mortality is historically high in Bangladesh. However, the country has achieved noteworthy progress in terms of reducing MMR by three quarters by 2015, as a part of its meeting the Millennium Development Goal 5A (MDG)[6]. According to a study based on

For peer review only - http://bmjopen.bmj.com/site/about/guidelines.xhtml

Bangladesh Maternal Mortality Surveys (BMMS), maternal mortality was the largest single cause of death (20%) for women aged 15–49 followed by malignancy and infectious diseases, and ranked third a decade later (14% deaths)[7]. Despite the continued progress, the country is lagging far behind in ensuring universal access to reproductive health (MDG 5B), and the rate of utilization of the basic MHS at the national level remains quite low[8]. According to Bangladesh Demographic and Health Survey (BDHS 2007), only about half of all mothers attended one or more antenatal visit and about one-fifth received at least one postnatal service. Mothers who do not attend ANC services are also more unlikely to deliver at health facilities and receive postnatal services[9], which increases the risk of pregnancy and childbirth related complications[5,9,10]. The rate of health facility delivery is also notably low in Bangladesh with three-quarters of all births occurring at home and merely one-fifth are attended by a skilled birth attendant (SBA)[10], which is far below the internationally agreed target (90% births to be attended by SBA by 2015) [11].

Previous studies have attempted to explore the barriers to the utilization of maternal healthcare services, some from demographic, economic[9,12-13] and some from sociocultural and behavioral perspectives[9,10,14-15]. Apart from the socioeconomic aspects, there is also a growing number of study emphasizing the role of women's decision-making autonomy on maternal health service utilization and pregnancy outcomes[16,17]. However, the results remain somewhat mixed as some researches stress on the importance of wife's autonomy on making decisions and some proposing that joint decision-making by husbands/partners and wives can produce better reproductive health outcomes than when one partner is left behind from decision-making tasks. In the perspective of Bangladesh however, involvement of husbands/partners in decision-making is particularly important because most families are male-headed and it is also the male figures who usually play the dominant role in important household decision making such as income expenditure and healthcare-related movement[17]. In South Asian countries including Bangladesh, gender discrimination and inequality remains a widespread phenomenon across various walks of life such as decision making autonomy, intra-household resource allocation, property rights and access to healthcare[18,19]. Women's autonomy is a multidimensional concept which is hard to represent in a single definition. In short, it conveys a set of discrete components or phenomena essential for ensuring that women can exercise their rights with full potential. Therefore, the aim of this study was to determine the association between women's decision-making power and utilization of maternal healthcare services among Bangladeshi women. For, this study, women's decision-making autonomy was measured across four different themes ranging from having a say in their own and children's healthcare decisions to household purchases and visiting family and

For peer review only - http://bmjopen.bmj.com/site/about/guidelines.xhtml

relatives. Data were sourced from the latest BDHS survey which provides a large scale quality data and representative of the general population.

Methods

The survey: BDHS 2014

This is a cross-sectional study based on data from the Bangladesh demographic and health survey conducted in 2014. The 2014 survey was the sixth to be conducted in the country. This is a nationally representative survey that included both urban and rural areas encompassing all seven administrative divisions—Dhaka, Rajshahi, Rangpur, Chittagong, Khulna, Barisal and Sylhet. A division is a collection of districts (zilas), and each district is further divided into administrative units (upazilas), which are further divided into urban and rural areas. Sample households were selected by a two-stage stratification technique. Firstly, 207 clusters in urban areas and 393 in rural areas were selected for 600 enumeration areas with proportional probability. In the second stage, on average 30 households were selected systematically from the enumeration areas. Finally, 17,989 households were selected for the survey of which 96% were interviewed successfully. Details on the survey and sampling technique are available in the final report.

Variables

Outcome variable: The outcome variables chosen for this study were three basic types of maternal healthcare services offered by the healthcare system in Bangladesh: 1) Antenatal care services, 2) facility delivery services, and 3) Post-delivery checkup services. Information on these topics were collected by face to face interview with the respondents. Women were asked the number of times they received Antenatal care (ANC), and the frequency ranged from ''0'' to ''20''. However, for this study, ANC was categorized as adequate (4/4+) and inadequate (<4) as per WHO recommendation which suggests at least four ANC attendance during pregnancy. Place of delivery was categorized as 'Facility delivery' and 'Delivery at home'. Facility delivery included delivery in public or private hospitals or clinics, NGO clinics. The third outcome variable- Post-delivery checkup services, was categorized as yes (For those who received any postnatal checkup) and no (For those who did not receive any postnatal checkup).

Explanatory variables of interest were women's decision making power on the following four themes: 1. Person who usually decides on respondent's health care, 2. Person who usually decides on large household purchases, 3. Final say on: Child health care, 4. Person who usually decides

For peer review only - http://bmjopen.bmj.com/site/about/guidelines.xhtml

on visits to family or relatives. In types of decision making tasks, a joint decision by women and their husband was highest. Possible answers were Respondent alone, Respondent and Husband/Partner jointly, Husband/Partner alone, and Other. The categories were collapsed into three by combining the last two into one (Husband/partner alone and Other).

The covariates included in the analysis were Age: 15-20/21-24/25-29/30+; Educational attainment: No education/Incomplete primary/Complete primary/Incomplete secondary/Complete secondary/Higher; Currently working: No/Yes; Wealth index: Poorer/Middle/Richer/Richest/Poorest; Parity: 1/2/3/3+.

Data analysis

Datasets were checked for missing values and outliers and weighted prior to analysis. Basic sociodemographic variables were described by descriptive statistics. Chi-square bivariate tests were performed to examine the group differences (Utilization vs Non- utilization of MCHs) for all the explanatory variables. The variables that showed significance at $p<=0.25$ in the bivariate tests were retained for final regression analysis. The association between utilization of MCHs and the independent variables was measured by binary logistic regression. Three separate regression models were run for each of the outcome variables. Results of the regression analysis were presented as adjusted odds ratios with corresponding 95% confidence intervals. The outcomes of the regression analysis were reported in terms of adjusted odds ratios (AOR) and corresponding 95% confidence intervals. Model fitness was verified by the Hosmer-Lemeshow goodness-of-fit test. All tests were two-tailed and was considered significant at 5%. Data were analysed using SPSS® version 22.

Ethics

All participants gave informed consent prior to taking part in the voluntary interview. The survey was approved by the ICF International Institutional Review Board (IRB) who is responsible for reviewing the procedures and questionnaires for standard DHS surveys.

Results

Population characteristics

Table 1 shows that majority of participants belonged to the youngest age groups of 15-20 years. About one-third of the women were from urban (32.5%) areas which is similar to the country's level scenario. 13.3% of the women had no formal education and 11.4% had completed primary level of education. Rate of illiteracy was high among rural women compared to their urban

For peer review only - http://bmjopen.bmj.com/site/about/guidelines.xhtml

counterparts (9.8 Vs 14.9%). Rate of completion of secondary was 7.5% and 11.8% had higher than secondary level education. Only about one-fifth of the women reported having an employment, and urban women had slightly higher rate of employment (22.9 Vs 19.0%) than rural women. Majority of the women belonged to the highest wealth quintile (20.5%) and a little less than one-fifth in the poorest wealth quintile (18.9%). A wide wealth disparity was observed between participants in urban and rural areas as 43.2% of the women in the highest wealth quintile were from urban areas compared with only 9.2% from rural areas. Two-fifth of the women had only one child and 14.2% had more than three children.

Table 1: Basic characteristics of the study population (N=4309), BDHS 2014.

Variables	N (%)	Urban	Rural
		1381 (32.5)	2873 (67.5)
**Age**			
15-20	1178 (27.7)	25.3	28.8
21-24	1144 (26.9)	28.7	26.0
25-29	1091 (25.6)	27.1	25.0
30+	841 (19.8)	18.9	20.2
**Educational attainment**			
No education	564 (13.3)	9.8	14.9
Incomplete primary	658 (15.5)	12.8	16.7
Complete primary	487 (11.4)	10.2	12.0
Incomplete secondary	1724 (40.5)	38.6	41.5
Complete secondary	319 (7.5)	8.9	6.8
Higher	502 (11.8)	19.6	8.0
**Currently working**			
No	3333 (78.3)	81.0	77.1
Yes	921 (21.7)	19.0	22.9
**Wealth index**			
Poorer	806 (18.9)	8.6	26.2
Middle	814 (19.1)	7.8	24.3
Richer	901 (21.2)	12.0	22.6
Richest	860 (20.2)	28.4	17.7
Poorest	873 (20.5)	43.2	9.2

For peer review only - http://bmjopen.bmj.com/site/about/guidelines.xhtml

**Parity**			
1	1700 (40.0)	44.6	37.7
2	1286 (30.2)	32.2	29.3
3	664 (15.6)	13.5	16.6
3+	604 (14.2)	9.7	16.4

Based on the availability of on the dataset, four types of decision making tasks were considered relevant to MCH in this study: 1. Person who usually decides on respondent's health care, 2. Person who usually decides on large household purchases, 3. Final say on: Child health care, 4. Person who usually decides on visits to family or relatives. For all types of decision making tasks, a joint decision by women and their husband was highest. Table 2 shows that frequency of having autonomy in all types of the decisions was lower among rural women except for final say on child's healthcare. In majority of the cases, decisions were made jointly by women and the husband/partner. Husbands/partners had notably higher rate of autonomy than women in making these decisions in both rural and urban areas.

Table 2: Women's household decision-making characteristics, BDHS 2014.

Types of decision making	Respondent alone		Respondent & husband/partner		Husband/partner alone/other	
	Urban	Rural	Urban	Rural	Urban	Rural
**Person who usually decides on respondent's health care**	12.2	12	51.3	47.2	36.5	40.9
**Person who usually decides on large household purchases**	7.1	5.4	53.4	46.5	39.5	48.0
**Final say on: Child's health care**	14.2	15.2	58.8	54.2	27.0	30.6
**Person who usually decides on visits to family or relatives**	8.3	7.4	53.4	47.3	38.3	45.2

For peer review only - http://bmjopen.bmj.com/site/about/guidelines.xhtml

Table 3 shows the prevalence of availing the three types of maternal healthcare services stratified by place of residency. Prevalence of ANC attendance, facility delivery and post-natal check-up were respectively 32.6%, 40.6% and 66.3% (Not shown in the table). Results of cross-tabulation show that the rate of utilization of these services were higher among urban women compared to their rural counterparts, higher among women ageing between 21-24 years, having incomplete secondary level schooling, living in the richest households, currently not working and had given birth only once. In majority of the cases, women who could make the decisions jointly with husband/partner were more like to enjoy the MCH services.

Table 3: **Percentage of women who reported utilizing three types of MCH across the explanatory variables, BDHS 2014.**

	**ANC**		**Facility delivery**		**Health check-up after birth**	
	**Urban (46.1)**	**Rural (26)**	**Urban (42.1)**	**Rural (67.7)**	**Urban (79.4)**	**Rural (60.0)**
**Age**						
15-20	29.0	26.7	28.2	27.1	29.4	26.0
21-24	27.9	27.4	29.2	25.2	27.6	24.4
25-29	19.6	15.8	19.6	17.5	18.7	19.5
30+	23.4	30.1	23.0	30.1	24.4	30.0
P-value	0.478	**0.005**	**0.051**	**0.101**	0.322	0.291
**Educational attainment**						
No education	5.2	6.8	5.0	6.8	8.6	10.9
Incomplete primary	7.7	12.2	9.3	10.1	11.5	14.7
Complete primary	7.4	8.6	6.3	9.7	8.8	11.3
Incomplete secondary	36.1	47.9	39.8	45.3	38.6	43.2
Complete secondary	11.0	10.7	11.1	10.9	9.9	8.6
Higher	32.7	13.9	28.5	17.2	22.7	11.4
P-value	**<0.001**	**<0.001**	**<0.001**	**<0.001**	**<0.001**	**<0.001**
**Wealth index**						
Poorest	5.2	15.6	3.1	12.6	5.7	20.0
Poorer	4.4	19.7	5.8	17.4	6.3	21.9
Middle	7.4	21.9	8.1	25.3	10.5	24.2

For peer review only - http://bmjopen.bmj.com/site/about/guidelines.xhtml

For peer review only

Richer	25.0	25.9	25.8	25.9	28.0	21.2
Richest	58.1	16.8	57.2	18.7	49.5	12.8
P-value	**<0.001**	**<0.001**	**<0.001**	**<0.001**	**<0.001**	**<0.001**
**Currently working**						
No	82.1	79.0	83.6	81.1	81.7	74.8
Yes	17.9	21.0	16.4	18.9	18.3	25.2
P-value	0.336	**0.157**	**0.004**	**<0.001**	**0.204**	**<0.001**
**Parity**						
1	50.1	44.0	50.2	49.1	46.3	42.2
2	34.7	32.1	33.0	29.2	33.3	28.7
3	10.0	14.7	11.0	13.8	12.4	15.5
4	5.2	9.2	5.8	8.0	8.0	13.7
P-value	**<0.001**	**<0.001**	**<0.001**	**<0.001**	**<0.001**	**<0.001**
**Decides on own health care**						
Alone	11.8	15.0	13.1	11.8	12.2	12.0
Jointly	53.7	49.3	51.8	49.6	52.6	48.1
Husband/other	34.5	35.7	35.0	38.5	35.1	39.9
P-value	**0.246**	**<0.001**	0.291	**0.165**	**0.096**	0.418
**Decides on large household purchases**						
Alone	7.8	6.8	8.1	5.5	7.7	5.8
Jointly	55.6	46.9	52.3	47.6	53.8	47.5
Husband/other	36.6	46.3	39.5	46.9	38.5	46.7
P-value	**0.108**	**0.117**	**0.189**	**0.144**	**0.149**	**0.006**
**Decides on Child health care**						
Alone	13.8	17.8	15.0	16.3	14.6	16.2
Jointly	61.9	55.1	59.4	54.9	59.4	54.9
Husband/other	24.3	27.1	25.5	28.8	26.0	28.9
P-value	**0.077**	**0.015**	**0.175**	**0.219**	**0.231**	**0.035**
**Decides on visits to family or relatives**						
**Alone**	8.6	8.0	9.9	7.2	8.3	7.7

For peer review only - http://bmjopen.bmj.com/site/about/guidelines.xhtml

**Jointly**	57.5	46.7	53.6	47.9	54.3	47.2
**Husband/other**	33.9	45.3	36.5	44.9	37.4	45.1
P-value	**0.007**	**0.140**	**0.029**	**0.102**	**0.075**	**0.031**

Association between decision making ability and utilization of MCH

Results of regression analysis on the association between decision making ability and utilization of MCH are presented in table 4.

In the urban areas, women who could decide their healthcare with husband/partner had 20% (95%CI=0.794-1.799) higher odds of attending at least four antenatal care compared to those who could make decisions alone. In the rural areas however, women who could make decisions alone were 35% (95%CI=0.464 0.897) less likely to do so. The odds of delivering at a health facility were 25% (95%CI=0.888-1.748) higher among rural women who made own health decisions jointly with husband/partner. Women in urban and rural areas had respectively 43% (0.941-2.169) and 28% (0.928-1.751) higher odds of receiving post-natal check-up when they made their health decisions jointly with husband/partner. Women in urban and rural areas who had less autonomy on deciding large household purchases were respectively 28% (95%CI=0.384-1.365) and 20% (95%CI=0.492-1.285) less likely to have at least 4 ANC visits. Rural women who had to decide on large household purchases with husband/partner had 15% (95%CI=0.547-1.332) lower odds of receiving post-natal check-up.

Having autonomy in deciding children's health care did not show noticeable impact on receiving ANC services. Odds of receiving post-natal check-up were respectively 22% (95%CI=0.503-1.212) and 31% (95%CI=0.501-0.946) lower and facility delivery respectively 11% (95%CI=0.574-1.413) and 12% (95%CI=0.577-1.266) lower among urban and rural women who had to make the decisions jointly with husband/partner. In urban areas, women who did not have the autonomy to decide on visiting family or relatives alone were 18% (95%CI=0.491-1.362) less likely to attend at least four antenatal visits. The odds of receiving post-natal check-up were respectively 32% (95%CI=0.760-2.311) and 11% (95%CI=0.757-1.636) higher among urban and rural women who could decide on visiting family or relatives jointly with husband/partner.

Table 4: Association between decision making ability and utilization of MCH in Bangladesh, BDHS 2014.

	Antenatal care	Delivery at a health facility	Health check-up after delivery

For peer review only - http://bmjopen.bmj.com/site/about/guidelines.xhtml

	OR (95%CI)		OR (95%CI)		OR (95%CI)	
	**Urban**	**Rural**	**Urban**	**Rural**	**Urban**	**Rural**
**Decides on own health care**						
Alone	1	1	1	1	1	1
Jointly	1.195 (0.794-1.799)	0.645 (0.464 0.897)	0.996 (0.606-1.327)	1.246 (0.888-1.748)	1.428 (0.941-2.169)	1.275 (0.928-1.751)
Husband/other	1.087 (0.806-1.750)	0.983 (0.635-1.227)	1.072 (0.731-1.572)	1.001 (0.703-1.424)	1.052 (0.662-1.671)	1.035 (0.815-1.579)
**Decides on large household purchases**						
Alone	1	1	1	1	1	1
Jointly	0.724 (0.384-1.365)	0.795 (0.492-1.285)	1.050 (0.621-1.776)	0.997 (0.629-1.581)	1.02 (0.251-1.745)	0.854 (0.547-1.332)
Husband/other	0.970 (0.361-1.444)	0.805 (0.493-1.315)	0.734 (0.420-1.282)	0.924 (0.587-1.455)	0.950 (0.617-1.935)	0.943 (0.548-1.497)
**Decides on Child health care**						
Alone	1	1	1	1	1	1
Jointly	0.978 (0.641-1.491)	0.983 (0.558-1.499)	0.897 (0.574-1.413)	0.884 (0.577-1.266)	0.781 (0.503-1.212)	0.688 (0.501-0.946)
Husband/other	1.100 (0.751-1.612)	0.930 (0.696-1.243)	1.079 (0.713-1.635)	0.945 (0.620-1.452)	0.898 (0.562-1.384)	0.949 (0.645-1.617)
**Decides on visits to family or relatives**						
Alone	1	1	1	1	1	1
Jointly	0.818 (0.491-1.362)	1.018 (0.645-1.727)	0.981 (0.664-0.178)	1.108 (0.776-1.729)	1.325 (0.760-2.311)	1.113 (0.757-1.636)

For peer review only - http://bmjopen.bmj.com/site/about/guidelines.xhtml

Husband/other	1.063 (0.701-1.928)	1.078 (0.709-1.640)	0.887 (0.421-1.121)	1.075 (0.711-1.625)	1.050 (0.819-1.567)	0.943 (0.645-1.378)

N.B. Adjusted for the variables found significant in the bivariate test in table 3.

Discussion and conclusion

Main findings

Based on a nationally representative data from Bangladesh demographic and health survey, this study explored the association between women's decision-making power and utilization of antenatal care, facility delivery and post-natal health check-up among adult non-pregnant women ageing between 15 and 49 years in Bangladesh. Our results show that the prevalence of antenatal care attendance, facility delivery and post-natal check-up were respectively 32.6%, 40.6% and 66.3% which indicates a considerable improvement compared to the previous estimates. In urban and rural areas respectively, the rate of attending at least four antenatal visits increased from 36.7% and 11.7% in 2004 (44.8% and 19.8% in 2011) to 46.1% and 26% in 2014[20]. Utilisation of health facility delivery increased from 12 % in 2004 (>29 % in 2011) to >40% in 2014[21], and postnatal check-up of mothers increased from 27.3% to >66% during the same period[22].

Compared to women who decided on their health care alone, those who decided jointly with husband/partner had higher likelihood of utilizing all three types of services (except for antenatal visits among rural women). However, women could decide large household purchases alone had higher likelihood of attending at least four antenatal visits. Similar association was observed for utilization of post-natal care among women in rural but not urban areas. Having decision-making autonomy on child's healthcare showed significant association with the utilization of facility delivery and postnatal check-ups but not antenatal visits. Having decision-making autonomy on visiting family/relatives showed significant association with the utilization of postnatal check-ups but not antenatal visits and facility delivery.

Comparison with existing literature

Results indicate that in majority of the cases decisions were made jointly followed by men alone and women alone. A previous study conducted on south Asian countries reported a similar situation that women's healthcare decision were made without their participation in Nepal (72.7%), Bangladesh (54.3%) and India (48.5%) [23]. Regarding the association between decision-

For peer review only - http://bmjopen.bmj.com/site/about/guidelines.xhtml

making autonomy and MHS utilization, comparison between the findings of the present study with the existing ones requires consideration of several important issues. Firstly, different studies uses different indicators of women's decision-making autonomy and different types of MHS. Moreover, some studies report involvement of various family members and not just women and husbands/partners. Regardless of that, our findings have consistent and conflicting points with previous ones. Low level of women's autonomy was found to be a contributing factor to poor maternal health service utilization in Nepal[24], India[25] , but not in Kenya[26]. In Ethiopia, decision-making autonomy on place of birth showed a positive association with utilization of institutional delivery[27].

While women's lack of decision-making autonomy can be attributed to poor utilization of MHS, it however should not be ignored that autonomy in certain circumstances can also result in less spousal communication and low male-involvement in reproductive care. Growing number of studies indicate that inadequate spousal communication and male-involvement in reproductive care are associated with poor reproductive and sexual health consequences, and recommend policies to promote spousal communication and cooperation for improved maternal health outcomes[28,29]. In Nepal for instance, economic autonomy among women was associated with lower likelihood of couple communication during pregnancy, while domestic decision-making autonomy was associated with both lower likelihood of intra-spousal communication during pregnancy and husband's presence at antenatal visits [30]. Husbands' involvement in antenatal care has been shown to have a positive influence on utilization of antenatal visits in Ethiopia[27]. Husbands' involvement was also associated with utilization of professional care during delivery in rural Bangladesh and India [31].

In light of the abovementioned discussion, it is suggestible that health projects aiming to improve the utilization of MHS should try to focus on women's autonomy and at the same time promote male involvement in women's reproductive care. A qualitative study on male participation in reproductive health in Bangladesh reported poor interaction between husband and wife regarding sexual reproductive health issues which makes it difficult for men to recognise the reproductive health issues of women[32]. The study also reported that men do not feel comfortable to take their wives to the health facility, which suggests the presence of complex social and cultural factors preventing effective spousal communication regarding reproductive health issues. In the traditionally male-dominated society in Bangladesh where male figures are usually involved in family decision making, excluding men from maternal health decision making issues could prevent men from making informed decision for their wives/partners.

For peer review only - http://bmjopen.bmj.com/site/about/guidelines.xhtml

For peer review only

This study has several limitations to declare. First, this study included only four aspects of women's decision making. Thus, the findings do no indicate women's overall mobility and empowerment but rather specifically focuses on a limited range of indicators. As the participants were only women, there remains a potential for bias/discordance regarding the level of autonomy enjoyed by women as this is to a large extent a subjective phenomenon. Arguably, collecting information from both men and women could generate more a reliable picture on women's mobility and empowerment. So the association between women's autonomy and health-care-service use may be underestimated when only women's reports are considered[30]. In addition, spousal autonomy is a complex concept and difficult to quantify and there is no universally agreed definition or tool for measurement. Last but not least, utilization status of MHS was reported by women and was not verified from medical records, and therefore subject to recall bias.

Abbreviations: BDHS: Bangladesh Demographic and Health Survey; MHS: Maternal health service utilization; MMR: Maternal mortality rate; SBA: Skilled birth attendant.

Conflict of Interest: None to declare.

Funding: This research was supported by the National Natural Science Foundation of China (71473097 and 71273097).

Data availability: Data are available from DHS website through registration.

Acknowledgement: Authors would like to express sincere thanks to DHS for distributing the datasets that made this study possible.

Authors' contributions: BG and DF contribute equally to this work. SFT, BG and ZCF designed the study. DF, BG and SFT conducted the analysis and wrote the paper. DF, SY, HZF, SG, OU participated in editing of the paper. All authors discussed the results and approved the revision of the final manuscript.

References

1. Benedict OA, Kontie MM, Martin S, *et al*. Distribution of causes of maternal mortality among different socio-demographic groups in Ghana; a descriptive study. *BMC Public Health* 2011; 11: 159.

For peer review only - http://bmjopen.bmj.com/site/about/guidelines.xhtml

2. Rush D. Nutrition and maternal mortality in the developing world. *Am J Clin Nutr* 2000;72(1):(suppl) 212S-240S.
3. Lale S, Chou D, Gemmill A, et al. Global causes of maternal death: a WHO systematic analysis. *Lancet Global Health* 2014;2:e323–33.
4. Kassebaum NJ, Bertozzi-Villa A, Coggeshall MS, et al. Global, regional, and national levels and causes of maternal mortality during 1990–2013: a systematic analysis for the Global Burden of Disease Study 2013. *Lancet* 2014; 384:980–1004.
5. Yaya S, Bishwajit G, Shah V. Wealth, education and urban–rural inequality and maternal healthcare service usage in Malawi. *BMJ Global Health* 2016; 1(2): e000085.
6. Marge K, Iqbal A, Malay KM, *et al*. Reducing Maternal Mortality and Improving Maternal Health: Bangladesh and MDG 5. *J Health Popul Nutr* 2008; 26(3): 280–294.
7. Quamrun N, Shams EA, Kanta J, *et al*. Causes of adult female deaths in Bangladesh: findings from two National Surveys. *BMC Public Health* 2015; 15: 911.
8. Shahabuddin AS, Delvaux T, Abouchadi S, *et al*. Utilization of maternal health services among adolescent women in Bangladesh: A scoping review of the literature. *Trop Med Int Health 2015*; 20(7):822-9.
9. Christiana RT, Cynthia LH, Peter H, *et al*. Why don't some women attend antenatal and postnatal care services? a qualitative study of community members' perspectives in Garut, Sukabumi and Ciamis districts of West Java Province, Indonesia. *BMC Pregnancy Childbirth* 2010; 10: 61.
10. Nazrul I, Mohammad TI, Yukie Y. Practices and determinants of delivery by skilled birth attendants in Bangladesh. *Reprod Health* 2014; 11: 86.
11. Sonya Crowe, Martin Utley, Anthony Costello and Christina Pagel. How many births in sub-Saharan Africa and South Asia will not be attended by a skilled birth attendant between 2011 and 2015? BMC Pregnancy and Childbirth, 2012, 12:4.
12. McNamee P, Ternent L, Hussein J. Barriers in accessing maternal healthcare: evidence from low-and middle-income countries. *Expert Rev Pharmacoecon Outcomes Res* 2009;9(1):41-8.
13. Ruhul A, Nirali MS, Stan B. Socioeconomic factors differentiating maternal and child health-seeking behavior in rural Bangladesh: A cross-sectional analysis. *Int J Equity Health* 2010; 9: 9.
14. Krishna KD, Yuba RP, Resham BK, *et al*. Barriers to Utilization of Antenatal Care Services in Eastern Nepal. *Front Public Health* 2015; 3: 197

For peer review only

For peer review only - http://bmjopen.bmj.com/site/about/guidelines.xhtml

15. David OA, Olufemi TO, Marianne V, *et al*. Determinants of health care seeking behaviour during pregnancy in Ogun State, Nigeria. *Reprod Health* 2016; 13(1): 32.

16. John KG, Bernard O, Alexander YS, *et al.* How intra-familial decision-making affects women's access to, and use of maternal healthcare services in Ghana: a qualitative study. *BMC Pregnancy Childbirth* 2015; 15: 173.

17. Story WT, Burgard SA. Couples' reports of household decision-making and the utilization of maternal health services in Bangladesh. *Soc Sci Med* 2012; 75(12):2403-11.

18. Fikree FF, Pasha O. Role of gender in health disparity: the South Asian context. *British Medical Journal* 2004; 328(7443):823–826.

19. Bishwajit G, Sarker S, Yaya S. Socio-cultural aspects of gender-based violence and its impacts on women's health in South Asia. *F1000Research* 2016; 5:802

20. Mohammad HP, Md NS, Jalal U, Ulrika E. Progress in the utilization of antenatal and delivery care services in Bangladesh: where does the equity gap lie? *BMC Pregnancy Childbirth* 2016; 16: 200.

21. Kamal SM. Preference for institutional delivery and caesarean sections in Bangladesh. *J Health Popul Nutr* 2013; 31(1):96-109.

22. Uzma S, Asiruddin Sk, Helal, *et al*. Immediate and Early Postnatal Care for Mothers and Newborns in Rural Bangladesh. *J Health Popul Nutr* 2006; 24(4): 508–518.

23. Senarath U, Gunawardena NS. Women's autonomy in decision making for health care in South Asia. *Asia Pac J Public Health* 2009; 21(2):137-43.

24. Ramesh Adhikari. Effect of Women's autonomy on maternal health service utilization in Nepal: a cross sectional study. *BMC Womens Health* 2016; 16: 26.

25. Mistry R, Galal O, Lu M. Women's autonomy and pregnancy care in rural India: a contextual analysis. *Soc Sci Med* 2009; 69(6):926-33.

26. Fotso JC, Ezeh AC, Essendi H. Maternal health in resource-poor urban settings: how does women's autonomy influence the utilization of obstetric care services? *Reprod Health* 2009; 16:6-9.

27. Biratu BT, Lindstrom DP. The influence of husbands' approval on women's use of prenatal care: results from Yirgalem and Jimma towns, south west Ethiopia. *Ethiopian Journal of Health Development* 2006; 20: pp. 84–92.

28. Mullany BC, Becker S, Hindin MJ. The impact of including husbands in antenatal health education services on maternal health practices in urban Nepal: results from a randomized controlled trial. *Health Education Research* 2007; 22: pp. 166–176.

For peer review only - http://bmjopen.bmj.com/site/about/guidelines.xhtml

29. Rasch V, Lyaruu MA. Unsafe abortion in Tanzania and the need for involving men in postabortion contraceptive counselling. *Studies in Family Planning* 2005; 36: pp. 301–310.

30. Allendorf K. Couples' reports of women's autonomy and health-care use in Nepal. *Stud Fam Plann* 2007; 38(1):35-46.

31. Deependra KT, Anke N. Women's autonomy and husbands' involvement in maternal health care in Nepal. *Social Science & Medicine* 2013; 93(9):1–10.

32. Shahjahan M, Kabir M. Why males in Bangladesh do not participate in reproductive health: lessons learned from focus group discussions. *Int Q Community Health Educ* 2006; 26(1):45-59.

For peer review only

STROBE 2007 (v4) Statement—Checklist of items that should be included in reports of *cross-sectional studies*

Section/Topic	Item #	Recommendation	Reported on page #
**Title and abstract**	1	(*a*) Indicate the study's design with a commonly used term in the title or the abstract	2
		(*b*) Provide in the abstract an informative and balanced summary of what was done and what was found	2
**Introduction**			
Background/rationale	2	Explain the scientific background and rationale for the investigation being reported	3
Objectives	3	State specific objectives, including any prespecified hypotheses	4
**Methods**			
Study design	4	Present key elements of study design early in the paper	5
Setting	5	Describe the setting, locations, and relevant dates, including periods of recruitment, exposure, follow-up, and data collection	5
Participants	6	(*a*) Give the eligibility criteria, and the sources and methods of selection of participants	5
Variables	7	Clearly define all outcomes, exposures, predictors, potential confounders, and effect modifiers. Give diagnostic criteria, if applicable	5
Data sources/ measurement	8*	For each variable of interest, give sources of data and details of methods of assessment (measurement). Describe comparability of assessment methods if there is more than one group	5,6
Bias	9	Describe any efforts to address potential sources of bias	*15*
Study size	10	Explain how the study size was arrived at	5
Quantitative variables	11	Explain how quantitative variables were handled in the analyses. If applicable, describe which groupings were chosen and why	6
Statistical methods	12	(*a*) Describe all statistical methods, including those used to control for confounding	6
		(*b*) Describe any methods used to examine subgroups and interactions	6
		(*c*) Explain how missing data were addressed	6
		(*d*) If applicable, describe analytical methods taking account of sampling strategy	
		(*e*) Describe any sensitivity analyses	
**Results**			

For peer review only

Participants	13*	(a) Report numbers of individuals at each stage of study—eg numbers potentially eligible, examined for eligibility, confirmed eligible, included in the study, completing follow-up, and analysed	5
		(b) Give reasons for non-participation at each stage	5
		(c) Consider use of a flow diagram	
Descriptive data	14*	(a) Give characteristics of study participants (eg demographic, clinical, social) and information on exposures and potential confounders	6-7
		(b) Indicate number of participants with missing data for each variable of interest	
Outcome data	15*	Report numbers of outcome events or summary measures	8
Main results	16	(*a*) Give unadjusted estimates and, if applicable, confounder-adjusted estimates and their precision (eg, 95% confidence interval). Make clear which confounders were adjusted for and why they were included	11-12
		(*b*) Report category boundaries when continuous variables were categorized	11-12
		(*c*) If relevant, consider translating estimates of relative risk into absolute risk for a meaningful time period	
Other analyses	17	Report other analyses done—eg analyses of subgroups and interactions, and sensitivity analyses	
**Discussion**			
Key results	18	Summarise key results with reference to study objectives	2
Limitations	19	Discuss limitations of the study, taking into account sources of potential bias or imprecision. Discuss both direction and magnitude of any potential bias	15
Interpretation	20	Give a cautious overall interpretation of results considering objectives, limitations, multiplicity of analyses, results from similar studies, and other relevant evidence	
Generalisability	21	Discuss the generalisability (external validity) of the study results	
**Other information**			2
Funding	22	Give the source of funding and the role of the funders for the present study and, if applicable, for the original study on which the present article is based	15

*Give information separately for cases and controls in case-control studies and, if applicable, for exposed and unexposed groups in cohort and cross-sectional studies.

Note: An Explanation and Elaboration article discusses each checklist item and gives methodological background and published examples of transparent reporting. The STROBE checklist is best used in conjunction with this article (freely available on the Web sites of PLoS Medicine at http://www.plosmedicine.org/, Annals of Internal Medicine at http://www.annals.org/, and Epidemiology at http://www.epidem.com/). Information on the STROBE Initiative is available at www.strobe-statement.org.

For peer review only

BMJ Open

Women's decision making autonomy and utilization of maternal healthcare services: results from the Bangladesh Demographic and Health Survey

Journal:	*BMJ Open*
Manuscript ID	bmjopen-2017-017142.R2
Article Type:	Research
Date Submitted by the Author:	27-Jul-2017

Complete List of Authors:	Ghose, Bishwajit; School of Medicine and Health Management, Tongji Medical College, Huazhong University of Science and Technology<br>Feng, Da; School of Medicine and Health Management, Tongji Medical College, Huazhong University of Science and Technology<br>Tang, Shangfeng; School of medicine and health management, Tongji medical college, Huazhong university of science and technology<br>Yaya, Sanni; School of International Development and Global Studies, University of Ottawa.<br>He, Zhifei; School of Medicine and Health Management, Tongji Medical College, Huazhong University of Science & Technology<br>Udenigwe, Ogochukwu; School of International Development and Global Studies, University of Ottawa.<br>Ghosh, Sharmistha; Department of Sociology, University of Dhaka<br>Feng, Zhanchun; School of Medicine and Health Management, Tongji Medical College, Huazhong University of Science and Technology

**Primary Subject Heading**:	Health services research
Secondary Subject Heading:	Public health
Keywords:	Bangladeshi women, Decision-making autonomy, Maternal healthcare services, Spousal cooperation

SCHOLARONE™ Manuscripts

Women's decision making autonomy and utilization of maternal healthcare services: results from the Bangladesh Demographic and Health Survey

Ghose Bishwajit[1†] Da Feng[1†] Shangfeng Tang[1*] Sanni Yaya[2]

Zhifei He[1] Ogochukwu Udenigwe[2] Sharmistha Ghosh[3] Zhanchun Feng[1]

[1] School of Medicine and Health Management, Tongji Medical College, Huazhong University of Science and Technology, Wuhan, China.

[2]School of International Development and Global Studies, University of Ottawa. Canada.

[3]Department of Sociology, University of Dhaka, Dhaka 1000, Bangladesh

Email addresses:

Ghose Bishwajit: brammaputram@gmail.com

Da Feng: fdnunu@163.com

Shangfeng Tang: sftang@hust.edu.cn

Sanni Yaya : sanni.yaya@uOttawa.ca

Zhifei He: houis123@163.com

Ogochukwu Udenigwe: ocudenigwe@gmail.com

Sharmistha Ghosh : aimidu.hust@yahoo.com

Zhanchun Feng :zcfeng@hust.edu.cn

† Both authors contributed equally to this work

***Correspondence:** Shangfeng Tang, sftang@hust.edu.cn

School of Medicine and Health Management, Tongji Medical College, Huazhong University of Science and Technology, Hangkong Road 13, Wuhan, Hubei, 430030 People's Republic of China; Email: sftang@hust.edu.cn

For peer review only - http://bmjopen.bmj.com/site/about/guidelines.xhtml

For peer review only

Abstract:

Objectives: The aim of this study was to determine the association between women's decision-making power and utilization of maternal healthcare services among Bangladeshi women.

Settings: This is a nationally representative survey that encompassed Dhaka, Rajshahi, Rangpur, Chittagong, Khulna, Barisal and Sylhet in Bangladesh. Sample households were selected by a two-stage stratification technique. Firstly, 207 clusters in urban areas and 393 in rural areas were selected for 600 enumeration areas with proportional probability. In the second stage, on average 30 households were selected systematically from the enumeration areas. Finally 17,989 household were selected for the survey of which 96% were interviewed successfully.

Participants: Cross-sectional data on 4309 non-pregnant women were collected from Bangladesh demographic and health survey 2014. Decision making status on respondent's own health care, large household purchases, having a say on child's health care, and visiting to family or relatives were included in the analysis.

Results: Prevalence of at least four antenatal attendance, facility delivery and post-natal check-up were respectively 32.6% (95%CI=31.2- 34), 40.6% (95%CI=39.13- 42.07) and 66.3% (95%CI=64.89- 67.71). Compared to women who could make decisions alone, women in the urban areas who had to decide on their healthcare with husband/partner had 20% (95%CI=0.794-1.799) higher odds of attending at least four antenatal visits and those in rural areas had 35% (95%CI=0.464-0.897) lower odds of attending at least four antenatal visits. Women in urban and rural areas had respectively 43% (95%CI=0.941-2.169) and 28% (95%CI=0.928-1.751) higher odds of receiving post-natal check-up when their health decisions were made jointly with their husband/partner.

Conclusion: Neither making decisions alone, nor deciding jointly with husband/partner was always positively associated with the utilization of all three types of MHS. This study concludes that better spousal cooperation on household and health issues could lead to higher utilization of MHS services.

Key terms: Bangladeshi women; Decision-making autonomy; Maternal healthcare services; Spousal cooperation.

For peer review only - http://bmjopen.bmj.com/site/about/guidelines.xhtml

Strengths and limitations

1. This is one of the few studies focusing on the correlation between women's decision making autonomy and maternal healthcare utilization in a South Asian country.
2. Based on data from Bangladesh Demographic and Health Survey 2014, this study provides the most recent scenario of the utilization of three key important components of maternal healthcare.
3. Regional differences were observed in the prevalence of decision making autonomy and utilization of maternal healthcare services. However, the association was not a strong indication regarding the importance of decision making autonomy for the uptake of maternal healthcare.
4. The survey was cross-sectional. Therefore, it cannot affirm any causal inference or direction of the association.

Introduction

There is a widespread consensus regarding the pivotal role of the utilization of maternal healthcare services (MHS) in reducing maternal and child mortality and promoting women's reproductive health. Maternal mortality refers to deaths caused by pregnancy or childbirth related complications. Since 2015, global maternal mortality rate (MMR) dropped by 44% at an average annual decline of 2.3%, however, it still remains the leading cause of death among adult women ageing between 15 and 49 years[1]. The burden of maternal mortality is also disproportionately skewed towards the developing countries[2.] The most important causes of maternal mortality in developing countries are unsafe abortion, haemorrhage, eclampsia and obstructed labour as they together account for nearly two-thirds of total maternal mortality globally[3,4]. Growing consensus suggests that a vast majority of these deaths are actually preventable simply by adopting the necessary precautions provisioned through basic maternal healthcare services (MHS)[3,5].

The burden of maternal mortality is historically high in Bangladesh. However, the country has achieved noteworthy progress in terms of reducing MMR by three quarters by 2015, as a part of its meeting the Millennium Development Goal 5A (MDG)[6]. According to a study based on

For peer review only - http://bmjopen.bmj.com/site/about/guidelines.xhtml

Bangladesh Maternal Mortality Surveys (BMMS), maternal mortality was the largest single cause of death (20%) for women aged 15–49 followed by malignancy and infectious diseases, and ranked third a decade later (14% deaths)[7]. Despite the continued progress, the country is lagging far behind in ensuring universal access to reproductive health (MDG 5B), and the rate of utilization of the basic MHS at the national level remains quite low[8]. According to Bangladesh Demographic and Health Survey (BDHS 2007), only about half of all mothers attended one or more antenatal visit and about one-fifth received at least one postnatal service. Mothers who do not attend ANC services are also more unlikely to deliver at health facilities and receive postnatal services[9], which increases the risk of pregnancy and childbirth related complications[5,9,10]. The rate of health facility delivery is also notably low in Bangladesh with three-quarters of all births occurring at home and merely one-fifth are attended by a skilled birth attendant (SBA)[10], which is far below the internationally agreed target (90% births to be attended by SBA by 2015) [11].

Previous studies have attempted to explore the barriers to the utilization of maternal healthcare services, some from demographic, economic[9,12-13] and some from sociocultural and behavioral perspectives[9,10,14-15]. Apart from the socioeconomic aspects, there is also a growing number of study emphasizing the role of women's decision-making autonomy on maternal health service utilization and pregnancy outcomes[16,17]. However, the results remain somewhat mixed as some researches stress on the importance of wife's autonomy on making decisions and some proposing that joint decision-making by husbands/partners and wives can produce better reproductive health outcomes than when one partner is left behind from decision-making tasks. In the perspective of Bangladesh however, involvement of husbands/partners in decision-making is particularly important because most families are male-headed and it is also the male figures who usually play the dominant role in important household decision making such as income expenditure and healthcare-related movement[17]. In South Asian countries including Bangladesh, gender discrimination and inequality remains a widespread phenomenon across various walks of life such as decision making autonomy, intra-household resource allocation, property rights and access to healthcare[18,19]. Women's autonomy is a multidimensional concept which is hard to represent in a single definition. In short, it conveys a set of discrete components or phenomena essential for ensuring that women can exercise their rights with full potential. Therefore, the aim of this study was to determine the association between women's decision-making power and utilization of maternal healthcare services among Bangladeshi women. For, this study, women's decision-making autonomy was measured across four different themes ranging from having a say in their own and children's healthcare decisions to household purchases and visiting family and

For peer review only - http://bmjopen.bmj.com/site/about/guidelines.xhtml
For peer review only

relatives. Data were sourced from the latest BDHS survey which provides a large scale quality data and representative of the general population.

Methods

The survey: BDHS 2014

This is a cross-sectional study based on data from the Bangladesh demographic and health survey conducted in 2014. The 2014 survey was the sixth to be conducted in the country. This is a nationally representative survey that included both urban and rural areas encompassing all seven administrative divisions—Dhaka, Rajshahi, Rangpur, Chittagong, Khulna, Barisal and Sylhet. A division is a collection of districts (zilas), and each district is further divided into administrative units (upazilas), which are further divided into urban and rural areas. Sample households were selected by a two-stage stratification technique. Firstly, 207 clusters in urban areas and 393 in rural areas were selected for 600 enumeration areas with proportional probability. In the second stage, on average 30 households were selected systematically from the enumeration areas. Finally, 17,989 households were selected for the survey of which 96% were interviewed successfully. Details on the survey and sampling technique are available in the final report.

Variables

Outcome variable: The outcome variables chosen for this study were three basic types of maternal healthcare services offered by the healthcare system in Bangladesh: 1) Antenatal care services, 2) facility delivery services, and 3) Post-delivery checkup services. Information on these topics were collected by face to face interview with the respondents. Women were asked the number of times they received Antenatal care (ANC), and the frequency ranged from ''0'' to ''20''. However, for this study, ANC was categorized as adequate (4/4+) and inadequate (<4) as per WHO recommendation which suggests at least four ANC attendance during pregnancy. Place of delivery was categorized as 'Facility delivery' and 'Delivery at home'. Facility delivery included delivery in public or private hospitals or clinics, NGO clinics. The third outcome variable- Post-delivery checkup services, was categorized as yes (For those who received any postnatal checkup) and no (For those who did not receive any postnatal checkup).

Explanatory variables of interest were women's decision making power on the following four themes: 1. Person who usually decides on respondent's health care, 2. Person who usually decides on large household purchases, 3. Final say on: Child health care, 4. Person who usually decides

For peer review only - http://bmjopen.bmj.com/site/about/guidelines.xhtml

For peer review only

on visits to family or relatives. In types of decision making tasks, a joint decision by women and their husband was highest. Possible answers were Respondent alone, Respondent and Husband/Partner jointly, Husband/Partner alone, and Other. The categories were collapsed into three by combining the last two into one (Husband/partner alone and Other).

The covariates included in the analysis were Age: 15-20/21-24/25-29/30+; Educational attainment: No education/Incomplete primary/Complete primary/Incomplete secondary/Complete secondary/Higher; Currently working: No/Yes; Wealth index: Poorer/Middle/Richer/Richest/Poorest; Parity: 1/2/3/3+.

Data analysis

Datasets were checked for missing values and outliers and weighted prior to analysis. Basic sociodemographic variables were described by descriptive statistics. Chi-square bivariate tests were performed to examine the group differences (Utilization vs Non- utilization of MCHs) for all the explanatory variables. The variables that showed significance at $p<=0.25$ in the bivariate tests were retained for final regression analysis. The association between utilization of MCHs and the independent variables was measured by binary logistic regression. Three separate regression models were run for each of the outcome variables. Results of the regression analysis were presented as adjusted odds ratios with corresponding 95% confidence intervals. The outcomes of the regression analysis were reported in terms of adjusted odds ratios (AOR) and corresponding 95% confidence intervals. Model fitness was verified by the Hosmer-Lemeshow goodness-of-fit test. All tests were two-tailed and was considered significant at 5%. Data were analysed using SPSS® version 22.

Ethics

All participants gave informed consent prior to taking part in the voluntary interview. The survey was approved by the ICF International Institutional Review Board (IRB) who is responsible for reviewing the procedures and questionnaires for standard DHS surveys.

Results

Population characteristics

Table 1 shows that majority of participants belonged to the youngest age groups of 15-20 years. About one-third of the women were from urban (32.5%) areas which is similar to the country's level scenario. 13.3% of the women had no formal education and 11.4% had completed primary level of education. Rate of illiteracy was high among rural women compared to their urban

For peer review only - http://bmjopen.bmj.com/site/about/guidelines.xhtml

counterparts (9.8 Vs 14.9%). Rate of completion of secondary was 7.5% and 11.8% had higher than secondary level education. Only about one-fifth of the women reported having an employment, and urban women had slightly higher rate of employment (22.9 Vs 19.0%) than rural women. Majority of the women belonged to the highest wealth quintile (20.5%) and a little less than one-fifth in the poorest wealth quintile (18.9%). A wide wealth disparity was observed between participants in urban and rural areas as 43.2% of the women in the highest wealth quintile were from urban areas compared with only 9.2% from rural areas. Two-fifth of the women had only one child and 14.2% had more than three children.

Table 1: Basic characteristics of the study population (N=4309), BDHS 2014.

Variables	N (%)	Urban	Rural
		1381 (32.5)	2873 (67.5)
**Age**			
15-20	1178 (27.7)	25.3	28.8
21-24	1144 (26.9)	28.7	26.0
25-29	1091 (25.6)	27.1	25.0
30+	841 (19.8)	18.9	20.2
**Educational attainment**			
No education	564 (13.3)	9.8	14.9
Incomplete primary	658 (15.5)	12.8	16.7
Complete primary	487 (11.4)	10.2	12.0
Incomplete secondary	1724 (40.5)	38.6	41.5
Complete secondary	319 (7.5)	8.9	6.8
Higher	502 (11.8)	19.6	8.0
**Currently working**			
No	3333 (78.3)	81.0	77.1
Yes	921 (21.7)	19.0	22.9
**Wealth index**			
Poorer	806 (18.9)	8.6	26.2
Middle	814 (19.1)	7.8	24.3
Richer	901 (21.2)	12.0	22.6
Richest	860 (20.2)	28.4	17.7
Poorest	873 (20.5)	43.2	9.2

For peer review only - http://bmjopen.bmj.com/site/about/guidelines.xhtml

**Parity**			
1	1700 (40.0)	44.6	37.7
2	1286 (30.2)	32.2	29.3
3	664 (15.6)	13.5	16.6
3+	604 (14.2)	9.7	16.4

Based on the availability of on the dataset, four types of decision making tasks were considered relevant to MCH in this study: 1. Person who usually decides on respondent's health care, 2. Person who usually decides on large household purchases, 3. Final say on: Child health care, 4. Person who usually decides on visits to family or relatives. For all types of decision making tasks, a joint decision by women and their husband was highest. Table 2 shows that frequency of having autonomy in all types of the decisions was lower among rural women except for final say on child's healthcare. In majority of the cases, decisions were made jointly by women and the husband/partner. Husbands/partners had notably higher rate of autonomy than women in making these decisions in both rural and urban areas.

Table 2: Women's household decision-making characteristics, BDHS 2014.

**Types of decision making**	**Respondent alone**		**Respondent & husband/partner**		**Husband/partner alone/other**	
	**Urban**	**Rural**	**Urban**	**Rural**	**Urban**	**Rural**
**Person who usually decides on respondent's health care**	12.2	12	51.3	47.2	36.5	40.9
**Person who usually decides on large household purchases**	7.1	5.4	53.4	46.5	39.5	48.0
**Final say on: Child's health care**	14.2	15.2	58.8	54.2	27.0	30.6
**Person who usually decides on visits to family or relatives**	8.3	7.4	53.4	47.3	38.3	45.2

For peer review only

For peer review only - http://bmjopen.bmj.com/site/about/guidelines.xhtml

Table 3 shows the prevalence of availing the three types of maternal healthcare services stratified by place of residency. Prevalence of ANC attendance, facility delivery and post-natal check-up were respectively 32.6%, 40.6% and 66.3% (Not shown in the table). Results of cross-tabulation show that the rate of utilization of these services were higher among urban women compared to their rural counterparts, higher among women ageing between 21-24 years, having incomplete secondary level schooling, living in the richest households, currently not working and had given birth only once. In majority of the cases, women who could make the decisions jointly with husband/partner were more like to enjoy the MCH services.

Table 3: **Percentage of women who reported utilizing three types of MCH across the explanatory variables, BDHS 2014.**

	**ANC**		**Facility delivery**		**Health check-up after birth**	
	**Urban (46.1)**	**Rural (26)**	**Urban (42.1)**	**Rural (67.7)**	**Urban (79.4)**	**Rural (60.0)**
**Age**						
15-20	29.0	26.7	28.2	27.1	29.4	26.0
21-24	27.9	27.4	29.2	25.2	27.6	24.4
25-29	19.6	15.8	19.6	17.5	18.7	19.5
30+	23.4	30.1	23.0	30.1	24.4	30.0
P-value	0.478	**0.005**	**0.051**	**0.101**	0.322	0.291
**Educational attainment**						
No education	5.2	6.8	5.0	6.8	8.6	10.9
Incomplete primary	7.7	12.2	9.3	10.1	11.5	14.7
Complete primary	7.4	8.6	6.3	9.7	8.8	11.3
Incomplete secondary	36.1	47.9	39.8	45.3	38.6	43.2
Complete secondary	11.0	10.7	11.1	10.9	9.9	8.6
Higher	32.7	13.9	28.5	17.2	22.7	11.4
P-value	**<0.001**	**<0.001**	**<0.001**	**<0.001**	**<0.001**	**<0.001**
**Wealth index**						
Poorest	5.2	15.6	3.1	12.6	5.7	20.0
Poorer	4.4	19.7	5.8	17.4	6.3	21.9
Middle	7.4	21.9	8.1	25.3	10.5	24.2

For peer review only - http://bmjopen.bmj.com/site/about/guidelines.xhtml

For peer review only

Richer	25.0	25.9	25.8	25.9	28.0	21.2
Richest	58.1	16.8	57.2	18.7	49.5	12.8
P-value	**<0.001**	**<0.001**	**<0.001**	**<0.001**	**<0.001**	**<0.001**
**Currently working**						
No	82.1	79.0	83.6	81.1	81.7	74.8
Yes	17.9	21.0	16.4	18.9	18.3	25.2
P-value	0.336	**0.157**	**0.004**	**<0.001**	**0.204**	**<0.001**
**Parity**						
1	50.1	44.0	50.2	49.1	46.3	42.2
2	34.7	32.1	33.0	29.2	33.3	28.7
3	10.0	14.7	11.0	13.8	12.4	15.5
4	5.2	9.2	5.8	8.0	8.0	13.7
P-value	**<0.001**	**<0.001**	**<0.001**	**<0.001**	**<0.001**	**<0.001**
**Decides on own health care**						
Alone	11.8	15.0	13.1	11.8	12.2	12.0
Jointly	53.7	49.3	51.8	49.6	52.6	48.1
Husband/other	34.5	35.7	35.0	38.5	35.1	39.9
P-value	**0.246**	**<0.001**	0.291	**0.165**	**0.096**	0.418
**Decides on large household purchases**						
Alone	7.8	6.8	8.1	5.5	7.7	5.8
Jointly	55.6	46.9	52.3	47.6	53.8	47.5
Husband/other	36.6	46.3	39.5	46.9	38.5	46.7
P-value	**0.108**	**0.117**	**0.189**	**0.144**	**0.149**	**0.006**
**Decides on Child health care**						
Alone	13.8	17.8	15.0	16.3	14.6	16.2
Jointly	61.9	55.1	59.4	54.9	59.4	54.9
Husband/other	24.3	27.1	25.5	28.8	26.0	28.9
P-value	**0.077**	**0.015**	**0.175**	**0.219**	**0.231**	**0.035**
**Decides on visits to family or relatives**						
**Alone**	8.6	8.0	9.9	7.2	8.3	7.7

For peer review only

For peer review only - http://bmjopen.bmj.com/site/about/guidelines.xhtml

**Jointly**	57.5	46.7	53.6	47.9	54.3	47.2
**Husband/other**	33.9	45.3	36.5	44.9	37.4	45.1
P-value	**0.007**	**0.140**	**0.029**	**0.102**	**0.075**	**0.031**

Association between decision making ability and utilization of MCH

Results of regression analysis on the association between decision making ability and utilization of MCH are presented in table 4.

In the urban areas, women who could decide their healthcare with husband/partner had 20% (95%CI=0.794-1.799) higher odds of attending at least four antenatal care compared to those who could make decisions alone. In the rural areas however, women who could make decisions alone were 35% (95%CI=0.464 0.897) less likely to do so. The odds of delivering at a health facility were 25% (95%CI=0.888-1.748) higher among rural women who made own health decisions jointly with husband/partner. Women in urban and rural areas had respectively 43% (0.941-2.169) and 28% (0.928-1.751) higher odds of receiving post-natal check-up when they made their health decisions jointly with husband/partner. Women in urban and rural areas who had less autonomy on deciding large household purchases were respectively 28% (95%CI=0.384-1.365) and 20% (95%CI=0.492-1.285) less likely to have at least 4 ANC visits. Rural women who had to decide on large household purchases with husband/partner had 15% (95%CI=0.547-1.332) lower odds of receiving post-natal check-up.

Having autonomy in deciding children's health care did not show noticeable impact on receiving ANC services. Odds of receiving post-natal check-up were respectively 22% (95%CI=0.503-1.212) and 31% (95%CI=0.501-0.946) lower and facility delivery respectively 11% (95%CI=0.574-1.413) and 12% (95%CI=0.577-1.266) lower among urban and rural women who had to make the decisions jointly with husband/partner. In urban areas, women who did not have the autonomy to decide on visiting family or relatives alone were 18% (95%CI=0.491-1.362) less likely to attend at least four antenatal visits. The odds of receiving post-natal check-up were respectively 32% (95%CI=0.760-2.311) and 11% (95%CI=0.757-1.636) higher among urban and rural women who could decide on visiting family or relatives jointly with husband/partner.

Table 4: Association between decision making ability and utilization of MCH in Bangladesh, BDHS 2014.

	Antenatal care	Delivery at a health facility	Health check-up after delivery

For peer review only

	OR (95%CI)		OR (95%CI)		OR (95%CI)	
	Urban	Rural	Urban	Rural	Urban	Rural
**Decides on own health care**						
Alone	1	1	1	1	1	1
Jointly	1.195 (0.794-1.799)	0.645 (0.464 0.897)	0.996 (0.606-1.327)	1.246 (0.888-1.748)	1.428 (0.941-2.169)	1.275 (0.928-1.751)
Husband/other	1.087 (0.806-1.750)	0.983 (0.635-1.227)	1.072 (0.731-1.572)	1.001 (0.703-1.424)	1.052 (0.662-1.671)	1.035 (0.815-1.579)
**Decides on large household purchases**						
Alone	1	1	1	1	1	1
Jointly	0.724 (0.384-1.365)	0.795 (0.492-1.285)	1.050 (0.621-1.776)	0.997 (0.629-1.581)	1.02 (0.251-1.745)	0.854 (0.547-1.332)
Husband/other	0.970 (0.361-1.444)	0.805 (0.493-1.315)	0.734 (0.420-1.282)	0.924 (0.587-1.455)	0.950 (0.617-1.935)	0.943 (0.548-1.497)
**Decides on Child health care**						
Alone	1	1	1	1	1	1
Jointly	0.978 (0.641-1.491)	0.983 (0.558-1.499)	0.897 (0.574-1.413)	0.884 (0.577-1.266)	0.781 (0.503-1.212)	0.688 (0.501-0.946)
Husband/other	1.100 (0.751-1.612)	0.930 (0.696-1.243)	1.079 (0.713-1.635)	0.945 (0.620-1.452)	0.898 (0.562-1.384)	0.949 (0.645-1.617)
**Decides on visits to family or relatives**						
Alone	1	1	1	1	1	1
Jointly	0.818 (0.491-1.362)	1.018 (0.645-1.727)	0.981 (0.664-0.178)	1.108 (0.776-1.729)	1.325 (0.760-2.311)	1.113 (0.757-1.636)

For peer review only - http://bmjopen.bmj.com/site/about/guidelines.xhtml

Husband/other	1.063 (0.701-1.928)	1.078 (0.709-1.640)	0.887 (0.421-1.121)	1.075 (0.711-1.625)	1.050 (0.819-1.567)	0.943 (0.645-1.378)

N.B. Adjusted for the variables found significant in the bivariate test in table 3.

Discussion and conclusion

Main findings

Based on a nationally representative data from Bangladesh demographic and health survey, this study explored the association between women's decision-making power and utilization of antenatal care, facility delivery and post-natal health check-up among adult non-pregnant women ageing between 15 and 49 years in Bangladesh. Our results show that the prevalence of antenatal care attendance, facility delivery and post-natal check-up were respectively 32.6%, 40.6% and 66.3% which indicates a considerable improvement compared to the previous estimates. In urban and rural areas respectively, the rate of attending at least four antenatal visits increased from 36.7% and 11.7% in 2004 (44.8% and 19.8% in 2011) to 46.1% and 26% in 2014[20]. Utilisation of health facility delivery increased from 12 % in 2004 (>29 % in 2011) to >40% in 2014[21], and postnatal check-up of mothers increased from 27.3% to >66% during the same period[22].

Compared to women who decided on their health care alone, those who decided jointly with husband/partner had higher likelihood of utilizing all three types of services (except for antenatal visits among rural women). However, women could decide large household purchases alone had higher likelihood of attending at least four antenatal visits. Similar association was observed for utilization of post-natal care among women in rural but not urban areas. Having decision-making autonomy on child's healthcare showed significant association with the utilization of facility delivery and postnatal check-ups but not antenatal visits. Having decision-making autonomy on visiting family/relatives showed significant association with the utilization of postnatal check-ups but not antenatal visits and facility delivery.

Comparison with existing literature

Results indicate that in majority of the cases decisions were made jointly followed by men alone and women alone. A previous study conducted on south Asian countries reported a similar situation that women's healthcare decision were made without their participation in Nepal (72.7%), Bangladesh (54.3%) and India (48.5%) [23]. Regarding the association between decision-

For peer review only - http://bmjopen.bmj.com/site/about/guidelines.xhtml

making autonomy and MHS utilization, comparison between the findings of the present study with the existing ones requires consideration of several important issues. Firstly, different studies uses different indicators of women's decision-making autonomy and different types of MHS. Moreover, some studies report involvement of various family members and not just women and husbands/partners. Regardless of that, our findings have consistent and conflicting points with previous ones. Low level of women's autonomy was found to be a contributing factor to poor maternal health service utilization in Nepal[24], India[25] , but not in Kenya[26]. In Ethiopia, decision-making autonomy on place of birth showed a positive association with utilization of institutional delivery[27].

While women's lack of decision-making autonomy can be attributed to poor utilization of MHS, it however should not be ignored that autonomy in certain circumstances can also result in less spousal communication and low male-involvement in reproductive care. Growing number of studies indicate that inadequate spousal communication and male-involvement in reproductive care are associated with poor reproductive and sexual health consequences, and recommend policies to promote spousal communication and cooperation for improved maternal health outcomes[28,29]. In Nepal for instance, economic autonomy among women was associated with lower likelihood of couple communication during pregnancy, while domestic decision-making autonomy was associated with both lower likelihood of intra-spousal communication during pregnancy and husband's presence at antenatal visits [30]. Husbands' involvement in antenatal care has been shown to have a positive influence on utilization of antenatal visits in Ethiopia[27]. Husbands' involvement was also associated with utilization of professional care during delivery in rural Bangladesh and India [31].

In light of the abovementioned discussion, it is suggestible that health projects aiming to improve the utilization of MHS should try to focus on women's autonomy and at the same time promote male involvement in women's reproductive care. A qualitative study on male participation in reproductive health in Bangladesh reported poor interaction between husband and wife regarding sexual reproductive health issues which makes it difficult for men to recognise the reproductive health issues of women[32]. The study also reported that men do not feel comfortable to take their wives to the health facility, which suggests the presence of complex social and cultural factors preventing effective spousal communication regarding reproductive health issues. In the traditionally male-dominated society in Bangladesh where male figures are usually involved in family decision making, excluding men from maternal health decision making issues could prevent men from making informed decision for their wives/partners.

For peer review only - http://bmjopen.bmj.com/site/about/guidelines.xhtml

This study has several limitations to declare. First, this study included only four aspects of women's decision making. Thus, the findings do no indicate women's overall mobility and empowerment but rather specifically focuses on a limited range of indicators. As the participants were only women, there remains a potential for bias/discordance regarding the level of autonomy enjoyed by women as this is to a large extent a subjective phenomenon. Arguably, collecting information from both men and women could generate more a reliable picture on women's mobility and empowerment. So the association between women's autonomy and health-care-service use may be underestimated when only women's reports are considered[30]. In addition, spousal autonomy is a complex concept and difficult to quantify and there is no universally agreed definition or tool for measurement. Last but not least, utilization status of MHS was reported by women and was not verified from medical records, and therefore subject to recall bias.

Abbreviations: BDHS: Bangladesh Demographic and Health Survey; MHS: Maternal health service utilization; MMR: Maternal mortality rate; SBA: Skilled birth attendant.

Conflict of Interest: None declared.

Funding: This research was supported by the National Natural Science Foundation of China (71473097 and 71273097).

Data availability: Data are available from DHS website through registration.

Acknowledgement: Authors would like to express sincere thanks to DHS for distributing the datasets that made this study possible.

Authors' contributions: BG and DF contribute equally to this work. SFT, BG and ZCF designed the study. DF, BG and SFT conducted the analysis and wrote the paper. DF, SY, HZF, SG, OU participated in editing of the paper. All authors discussed the results and approved the revision of the final manuscript.

References

1. Benedict OA, Kontie MM, Martin S, *et al*. Distribution of causes of maternal mortality among different socio-demographic groups in Ghana; a descriptive study. *BMC Public Health* 2011; 11: 159.

For peer review only - http://bmjopen.bmj.com/site/about/guidelines.xhtml

2. Rush D. Nutrition and maternal mortality in the developing world. *Am J Clin Nutr* 2000;72(1):(suppl) 212S-240S.
3. Lale S, Chou D, Gemmill A, et al. Global causes of maternal death: a WHO systematic analysis. *Lancet Global Health* 2014;2:e323–33.
4. Kassebaum NJ, Bertozzi-Villa A, Coggeshall MS, et al. Global, regional, and national levels and causes of maternal mortality during 1990–2013: a systematic analysis for the Global Burden of Disease Study 2013. *Lancet* 2014; 384:980–1004.
5. Yaya S, Bishwajit G, Shah V. Wealth, education and urban–rural inequality and maternal healthcare service usage in Malawi. *BMJ Global Health* 2016; 1(2): e000085.
6. Marge K, Iqbal A, Malay KM, *et al*. Reducing Maternal Mortality and Improving Maternal Health: Bangladesh and MDG 5. *J Health Popul Nutr* 2008; 26(3): 280–294.
7. Quamrun N, Shams EA, Kanta J, *et al*. Causes of adult female deaths in Bangladesh: findings from two National Surveys. *BMC Public Health* 2015; 15: 911.
8. Shahabuddin AS, Delvaux T, Abouchadi S, *et al*. Utilization of maternal health services among adolescent women in Bangladesh: A scoping review of the literature. *Trop Med Int Health 2015*; 20(7):822-9.
9. Christiana RT, Cynthia LH, Peter H, *et al*. Why don't some women attend antenatal and postnatal care services? a qualitative study of community members' perspectives in Garut, Sukabumi and Ciamis districts of West Java Province, Indonesia. *BMC Pregnancy Childbirth* 2010; 10: 61.
10. Nazrul I, Mohammad TI, Yukie Y. Practices and determinants of delivery by skilled birth attendants in Bangladesh. *Reprod Health* 2014; 11: 86.
11. Sonya Crowe, Martin Utley, Anthony Costello and Christina Pagel. How many births in sub-Saharan Africa and South Asia will not be attended by a skilled birth attendant between 2011 and 2015? BMC Pregnancy and Childbirth, 2012, 12:4.
12. McNamee P, Ternent L, Hussein J. Barriers in accessing maternal healthcare: evidence from low-and middle-income countries. *Expert Rev Pharmacoecon Outcomes Res* 2009;9(1):41-8.
13. Ruhul A, Nirali MS, Stan B. Socioeconomic factors differentiating maternal and child health-seeking behavior in rural Bangladesh: A cross-sectional analysis. *Int J Equity Health* 2010; 9: 9.
14. Krishna KD, Yuba RP, Resham BK, *et al*. Barriers to Utilization of Antenatal Care Services in Eastern Nepal. *Front Public Health* 2015; 3: 197

For peer review only

15. David OA, Olufemi TO, Marianne V, *et al*. Determinants of health care seeking behaviour during pregnancy in Ogun State, Nigeria. *Reprod Health* 2016; 13(1): 32.
16. John KG, Bernard O, Alexander YS, *et al.* How intra-familial decision-making affects women's access to, and use of maternal healthcare services in Ghana: a qualitative study. *BMC Pregnancy Childbirth* 2015; 15: 173.
17. Story WT, Burgard SA. Couples' reports of household decision-making and the utilization of maternal health services in Bangladesh. *Soc Sci Med* 2012; 75(12):2403-11.
18. Fikree FF, Pasha O. Role of gender in health disparity: the South Asian context. *British Medical Journal* 2004; 328(7443):823–826.
19. Bishwajit G, Sarker S, Yaya S. Socio-cultural aspects of gender-based violence and its impacts on women's health in South Asia. *F1000Research* 2016; 5:802
20. Mohammad HP, Md NS, Jalal U, Ulrika E. Progress in the utilization of antenatal and delivery care services in Bangladesh: where does the equity gap lie? *BMC Pregnancy Childbirth* 2016; 16: 200.
21. Kamal SM. Preference for institutional delivery and caesarean sections in Bangladesh. *J Health Popul Nutr* 2013; 31(1):96-109.
22. Uzma S, Asiruddin Sk, Helal, *et al*. Immediate and Early Postnatal Care for Mothers and Newborns in Rural Bangladesh. *J Health Popul Nutr* 2006; 24(4): 508–518.
23. Senarath U, Gunawardena NS. Women's autonomy in decision making for health care in South Asia. *Asia Pac J Public Health* 2009; 21(2):137-43.
24. Ramesh Adhikari. Effect of Women's autonomy on maternal health service utilization in Nepal: a cross sectional study. *BMC Womens Health* 2016; 16: 26.
25. Mistry R, Galal O, Lu M. Women's autonomy and pregnancy care in rural India: a contextual analysis. *Soc Sci Med* 2009; 69(6):926-33.
26. Fotso JC, Ezeh AC, Essendi H. Maternal health in resource-poor urban settings: how does women's autonomy influence the utilization of obstetric care services? *Reprod Health* 2009; 16:6-9.
27. Biratu BT, Lindstrom DP. The influence of husbands' approval on women's use of prenatal care: results from Yirgalem and Jimma towns, south west Ethiopia. *Ethiopian Journal of Health Development* 2006; 20: pp. 84–92.
28. Mullany BC, Becker S, Hindin MJ. The impact of including husbands in antenatal health education services on maternal health practices in urban Nepal: results from a randomized controlled trial. *Health Education Research* 2007; 22: pp. 166–176.

For peer review only - http://bmjopen.bmj.com/site/about/guidelines.xhtml

29. Rasch V, Lyaruu MA. Unsafe abortion in Tanzania and the need for involving men in postabortion contraceptive counselling. *Studies in Family Planning* 2005; 36: pp. 301–310.

30. Allendorf K. Couples' reports of women's autonomy and health-care use in Nepal. *Stud Fam Plann* 2007; 38(1):35-46.

31. Deependra KT, Anke N. Women's autonomy and husbands' involvement in maternal health care in Nepal. *Social Science & Medicine* 2013; 93(9):1–10.

32. Shahjahan M, Kabir M. Why males in Bangladesh do not participate in reproductive health: lessons learned from focus group discussions. *Int Q Community Health Educ* 2006; 26(1):45-59.

For peer review only

For peer review only - http://bmjopen.bmj.com/site/about/guidelines.xhtml

STROBE 2007 (v4) Statement—Checklist of items that should be included in reports of *cross-sectional studies*

Section/Topic	Item #	Recommendation	Reported on page #
**Title and abstract**	1	(*a*) Indicate the study's design with a commonly used term in the title or the abstract	2
		(*b*) Provide in the abstract an informative and balanced summary of what was done and what was found	2
**Introduction**			
Background/rationale	2	Explain the scientific background and rationale for the investigation being reported	3
Objectives	3	State specific objectives, including any prespecified hypotheses	4
**Methods**			
Study design	4	Present key elements of study design early in the paper	5
Setting	5	Describe the setting, locations, and relevant dates, including periods of recruitment, exposure, follow-up, and data collection	5
Participants	6	(*a*) Give the eligibility criteria, and the sources and methods of selection of participants	5
Variables	7	Clearly define all outcomes, exposures, predictors, potential confounders, and effect modifiers. Give diagnostic criteria, if applicable	5
Data sources/ measurement	8*	For each variable of interest, give sources of data and details of methods of assessment (measurement). Describe comparability of assessment methods if there is more than one group	5,6
Bias	9	Describe any efforts to address potential sources of bias	*15*
Study size	10	Explain how the study size was arrived at	5
Quantitative variables	11	Explain how quantitative variables were handled in the analyses. If applicable, describe which groupings were chosen and why	6
Statistical methods	12	(*a*) Describe all statistical methods, including those used to control for confounding	6
		(*b*) Describe any methods used to examine subgroups and interactions	6
		(*c*) Explain how missing data were addressed	6
		(*d*) If applicable, describe analytical methods taking account of sampling strategy	
		(*e*) Describe any sensitivity analyses	
**Results**			

For peer review only

Participants	13*	(a) Report numbers of individuals at each stage of study—eg numbers potentially eligible, examined for eligibility, confirmed eligible, included in the study, completing follow-up, and analysed	5
		(b) Give reasons for non-participation at each stage	5
		(c) Consider use of a flow diagram	
Descriptive data	14*	(a) Give characteristics of study participants (eg demographic, clinical, social) and information on exposures and potential confounders	6-7
		(b) Indicate number of participants with missing data for each variable of interest	
Outcome data	15*	Report numbers of outcome events or summary measures	8
Main results	16	(*a*) Give unadjusted estimates and, if applicable, confounder-adjusted estimates and their precision (eg, 95% confidence interval). Make clear which confounders were adjusted for and why they were included	11-12
		(*b*) Report category boundaries when continuous variables were categorized	11-12
		(*c*) If relevant, consider translating estimates of relative risk into absolute risk for a meaningful time period	
Other analyses	17	Report other analyses done—eg analyses of subgroups and interactions, and sensitivity analyses	
**Discussion**			
Key results	18	Summarise key results with reference to study objectives	2
Limitations	19	Discuss limitations of the study, taking into account sources of potential bias or imprecision. Discuss both direction and magnitude of any potential bias	15
Interpretation	20	Give a cautious overall interpretation of results considering objectives, limitations, multiplicity of analyses, results from similar studies, and other relevant evidence	
Generalisability	21	Discuss the generalisability (external validity) of the study results	
**Other information**			2
Funding	22	Give the source of funding and the role of the funders for the present study and, if applicable, for the original study on which the present article is based	15

*Give information separately for cases and controls in case-control studies and, if applicable, for exposed and unexposed groups in cohort and cross-sectional studies.

Note: An Explanation and Elaboration article discusses each checklist item and gives methodological background and published examples of transparent reporting. The STROBE checklist is best used in conjunction with this article (freely available on the Web sites of PLoS Medicine at http://www.plosmedicine.org/, Annals of Internal Medicine at http://www.annals.org/, and Epidemiology at http://www.epidem.com/). Information on the STROBE Initiative is available at www.strobe-statement.org.

For peer review only
